# Continuous Cropping Differentially Regulates Nitrogen Metabolism and Nodulation in Soybean Cultivars with Contrasting Continuous Cropping Tolerance Under Varied Nitrogen Levels

**DOI:** 10.3390/plants15182839

**Published:** 2026-09-17

**Authors:** Wenbo Liu, Fangang Meng, Demin Rao, Xingdong Yao

**Affiliations:** 1 Hebei Key Laboratory of Crop Stress Biology, College of Agronomy and Biotechnology, Hebei Normal University of Science & Technology, Qinhuangdao 066000, China; lwbsynydx@163.com; 2 Soybean Research Institute, Jilin Academy of Agricultural Sciences, National Engineering Research Center of Soybean, Changchun 130033, China; mengfg2013@163.com; 3 Soybean Research Institute, Shenyang Agricultural University, Shenyang 110866, China

**Keywords:** continuous cropping, soybean, nitrogen level, nitrogen metabolism, nodulation

## Abstract

Continuous cropping obstacles severely restrict soybean production; nitrogen application can alleviate continuous cropping obstacles. However, it remains unclear how nitrogen metabolism and nodulation differ between soybean cultivars with contrasting continuous cropping tolerance under varied nitrogen levels. In this study, two soybean cultivars, Liaodou 14 (L14, continuous-cropping-tolerant) and Liaodou 10 (L10, continuous-cropping-sensitive), were cultivated in a sand culture system. Three nitrogen levels, low (LN), medium (MN), and high (HN), were set with three soybean soil extract treatments, namely continuous cropping (CC), continuous cropping + sodium orthovanadate (CS), and crop rotation (CR). Key nitrogen metabolic enzyme activities and gene expression in leaves and fine roots, nodule number, nitrogenase activity, and nitrogen content were systematically determined. The results show that the CC treatment significantly inhibited nitrogen metabolism enzyme activities, gene expression, and nitrogen accumulation, whereas the HN treatment partially alleviated the growth-related inhibitory effects induced by continuous cropping, although HN treatment did not fully restore symbiotic nitrogen fixation function. Under the CC treatment, L14 exhibited superior performance over L10 for most measured parameters, except for GDH activity and *GmNADH-GOGAT* expression. Specifically, the leaf NR activity of L14 was 8.51% and 8.47% higher than that of L10 at the R1 and R6 growth stages, respectively. The maximum nodule number was observed under the HN treatment, while the highest nitrogenase activity occurred under the LN treatment. Most indicators under the CS treatment showed intermediate values between the CC and CR treatments. Collectively, through optimized nitrogen management, continuous-cropping-tolerant cultivars maintain higher enzyme activities, stable gene expression and efficient nitrogen translocation and allocation, potentially alleviating continuous cropping obstacles and improving yield potential.

## 1. Introduction

Soybean (*Glycine max* (L.) Merr.) is an important food and oil crop in China, but domestic production falls far short of consumption demand [1]. In Northeast China, due to limitations in arable land resources, continuous cropping of soybean is widespread. Long-term continuous cropping not only leads to soil nutrient depletion and deterioration of physicochemical properties but also directly inhibits plant growth and development, ultimately severely affecting yield and seed quality [2]. Although soybean can fix atmospheric nitrogen through symbiosis with rhizobia, the amount of fixed biological nitrogen is generally insufficient to meet the nitrogen demand for high-yield cultivation [3,4]. Therefore, the rational application of nitrogen fertilizer is both an important measure to ensure high yield and a potential approach to alleviate continuous cropping obstacles. However, the physiological mechanisms by which different nitrogen levels alleviate continuous cropping obstacles in soybean remain unclear.

Nodules are the primary sites of biological nitrogen fixation in leguminous crops [5] and can supply 50–60% of the total nitrogen requirement of soybean [3]. Nodule formation and function are regulated by multiple factors, including host genotype, nitrogen supply, and soil environment [6]. High concentrations of nitrate nitrogen inhibit nodule growth, development, and nitrogenase activity [7], while an appropriate nitrogen supply favors the establishment of the nodulation and nitrogen fixation system [3]. Different nitrogen fertilizer treatments significantly affect the nodule number of soybean and alter the peak period of the nodule number; moreover, under the same nitrogen fertilizer treatment, different soybean cultivars exhibit significantly different nodule numbers, based on genotype [8]. Under continuous cropping, due to the accumulation of soil pathogens and autotoxic substances, nodule formation and nitrogen fixation function are further inhibited. However, the physiological mechanisms by which nitrogen levels regulate nodulation and nitrogen fixation in soybean under continuous cropping stress still require in-depth investigation.

The nitrogen fertilizer utilization efficiency of soybean depends on the activities of key enzymes involved in nitrogen metabolism and assimilation, as well as the regulation of related gene expression. Nitrate reductase (NR) is the rate-limiting enzyme in nitrate nitrogen assimilation, and its activity directly determines the efficiency of nitrate reduction to nitrite [9]. The GS-GOGAT cycle, composed of glutamine synthetase (GS) and glutamate synthase (GOGAT), represents the predominant pathway for ammonium assimilation in higher plants [10], and glutamate dehydrogenase (GDH) is involved under specific conditions [11,12]. The activities of NR, GS, GOGAT, and GDH, together with the expression levels of nitrogen metabolism-related genes, collectively determine the nitrogen fertilizer utilization efficiency of soybean and consequently affect yield formation. However, there is still a lack of systematic studies on the responses of key nitrogen metabolism enzyme activities and their gene expression to nitrogen levels in soybean cultivars with different continuous cropping tolerances.

Soybean nitrogen absorption and utilization efficiency are influenced by genotype [13] and nitrogen source form [14], with significant variation among cultivars [15]. Under continuous cropping, the mechanisms underlying the differences in nitrogen absorption, assimilation, and utilization among soybean cultivars with contrasting tolerance remain unclear. Root plasma membrane H^+^-ATPase is considered the “master enzyme” of plant cells [16]. It not only transports ions and organic compounds across the plasma membrane via channels and carriers but also participates in diverse physiological processes essential for plant growth, including salt tolerance, intracellular pH regulation, and cell growth and expansion [17]. Studies have shown that sodium orthovanadate (Na_3_VO_4_) can significantly inhibit root plasma membrane H^+^-ATPase activity, thereby weakening the transmembrane electrochemical potential and suppressing the transport of organic compounds, including the uptake of harmful substances by soybean roots [18]. Nevertheless, sodium orthovanadate acts as a broad-spectrum inhibitor of phosphatases and H^+^-ATPase, which can interfere with multiple physiological processes. In this study, a sand culture system was employed to precisely regulate nutrient supply, soil extracts were added to simulate a continuous cropping environment, and sodium orthovanadate was included as an exogenous chemical treatment, with the aim of more accurately evaluating nitrogen metabolism capacity in soybean cultivars differing in continuous cropping tolerance.

Despite the recognized role of nitrogen fertilizer in alleviating continuous cropping obstacles in soybean, the differential regulatory mechanisms by which nitrogen supply levels affect nitrogen metabolism and nodulation capacity in cultivars with contrasting continuous cropping tolerance remain poorly understood. In particular, the intrinsic relationships among key nitrogen assimilation enzyme activities, symbiotic nitrogen fixation efficiency, and nitrogen accumulation under continuous cropping stress, as well as their responses to nitrogen supply and their variation between cultivars, require further investigation. To address this issue, we selected two soybean cultivars with different tolerances to continuous cropping as materials, with three nitrogen levels and three soil extract treatments. Nitrogen metabolism enzyme activities and the expression levels of related key genes, nodule number, and nitrogenase activity, as well as nitrogen accumulation and distribution, were systematically measured. The objectives were to (i) characterize differential responses of nitrogen metabolism capacity to varying nitrogen levels between cultivars with distinct continuous cropping tolerances; (ii) clarify the physiological mechanisms underlying efficient nitrogen utilization in continuous-cropping-tolerant soybean cultivars; and (iii) establish a theoretical foundation for optimizing nitrogen fertilizer management and promoting the adoption of continuous-cropping-tolerant cultivars in continuous cropping systems.

## 2. Results

### 2.1. Nitrogen Metabolism Enzyme Activities

**Figure 1:**
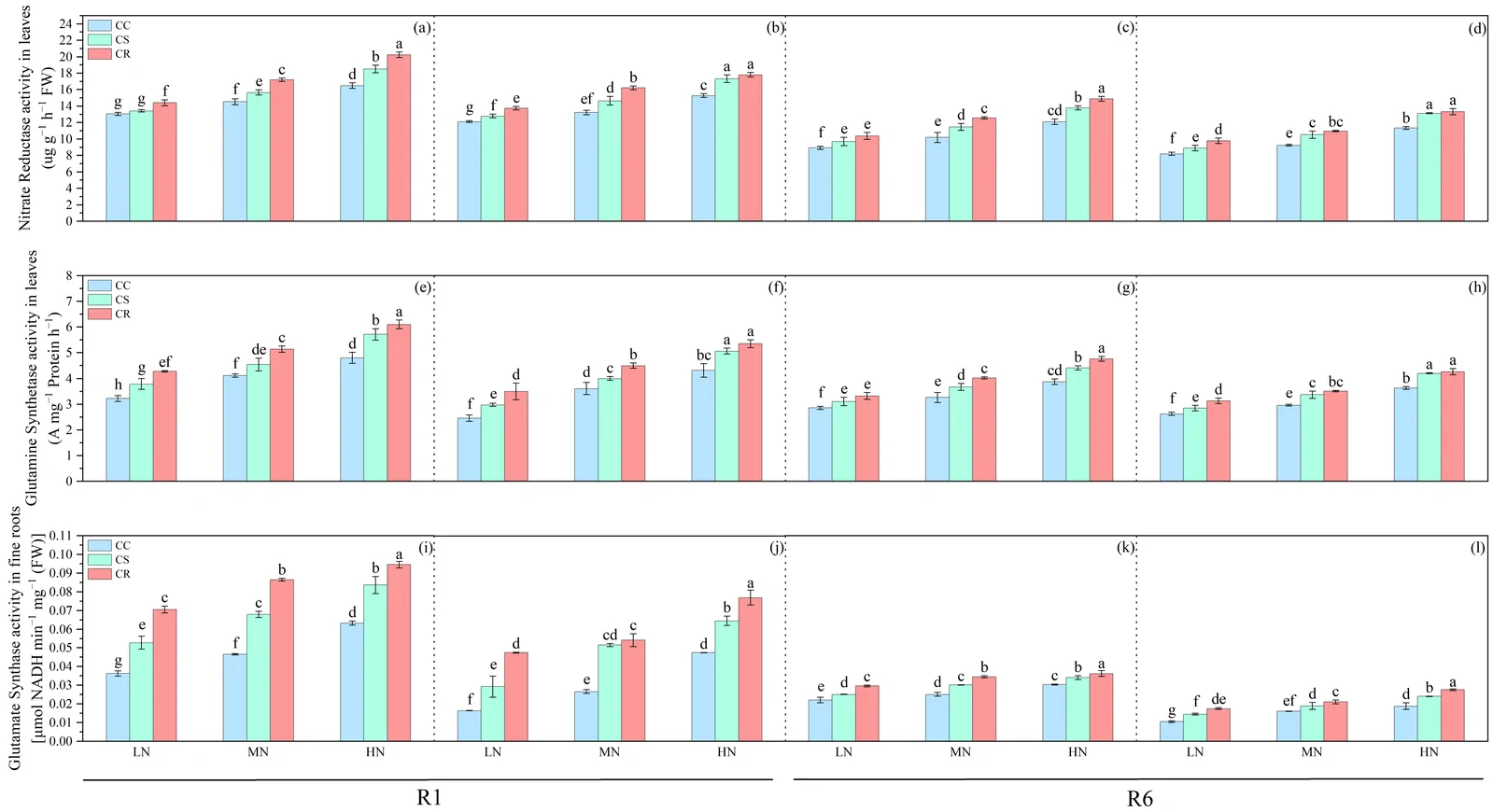
Effects of nitrogen levels on nitrogen metabolism enzymes in leaves and fine roots of soybean cultivars with different continuous cropping tolerance. Different lowercase letters above bars indicate significant differences among treatments within each cultivar at *p* < 0.05. Abbreviations: R1, initial flowering growth stage; R6, full seed growth stage; LN, low nitrogen; MN, medium nitrogen; HN, high nitrogen; CC, soybean continuous cropping soil extract; CS, soybean continuous cropping soil extract + sodium orthovanadate (Na_3_VO_4_); CR, soybean crop rotation soil extract. Panels (**a**,**b**,**e**,**f**,**i**,**j**) represent the R1 growth stage; panels (**c**,**d**,**g**,**h**,**k**,**l**) represent the R6 growth stage. Panels (**a**,**c**,**e**,**g**,**i**,**k**) represent Liaodou 14 (L14); panels (**b**,**d**,**f**,**h**,**j**,**l**) represent Liaodou 10 (L10).

A three-way ANOVA revealed that cultivar, nitrogen level, and soil extract treatment had varying degrees of influence on nitrogen metabolism enzyme activities in soybean leaves and fine roots, with significant main effects observed for most enzyme activities (Table S3). The soybean continuous cropping soil extract (CC) treatment significantly reduced the activities of nitrate reductase (NR), glutamine synthetase (GS), and glutamate synthase (GOGAT) in the two cultivars (*p* < 0.05). At the initial flowering growth stage (R1), NR, GS, and GOGAT activities of Liaodou 10 (L10) were lower than that of Liaodou 14 (L14) across all nitrogen levels; at the full seed growth stage (R6), NR, GS, and GOGAT activities in leaves showed significantly less reduction in L14. The high-nitrogen (HN) treatment alleviated the inhibitory effects of continuous cropping: at the R1 growth stage, NR activity in the leaves of L14 and L10 under the HN treatment reached 81.49% and 85.82% of the soybean crop rotation soil extract (CR) treatment, respectively; at the R6 growth stage, it reached 81.24% and 85.07% of the CR treatment, respectively. GS and GOGAT activities showed similar trends (Figure 1 and Table S6). The soybean continuous cropping soil extract + sodium orthovanadate (CS) treatment exhibited intermediate enzyme activities between the CC and CR treatments. Nitrite reductase (NiR) activity showed a similar trend to NR, GS, and GOGAT, whereas glutamate dehydrogenase (GDH) activity exhibited the opposite pattern (Table S6).

### 2.2. Expression of Nitrogen Metabolism-Related Genes

**Figure 2:**
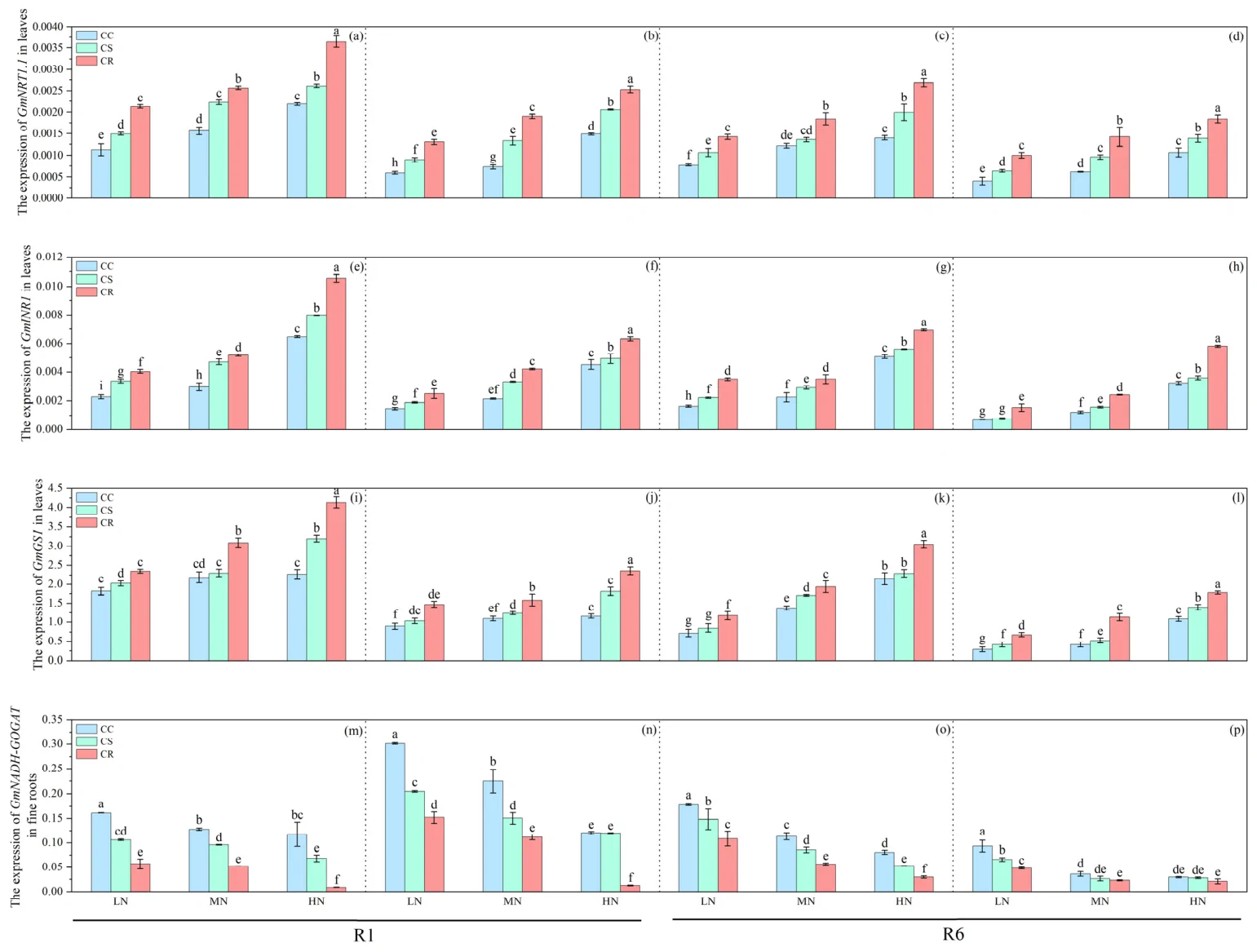
Effects of nitrogen levels on relative expression levels of key nitrogen metabolism-related genes in leaves and fine roots of soybean cultivars with different continuous cropping tolerance. Different lowercase letters above bars indicate significant differences among treatments within each cultivar at *p* < 0.05. Abbreviations: R1, initial flowering growth stage; R6, full seed growth stage; LN, low nitrogen; MN, medium nitrogen; HN, high nitrogen; CC, soybean continuous cropping soil extract; CS, soybean continuous cropping soil extract + sodium orthovanadate (Na_3_VO_4_); CR, soybean crop rotation soil extract. Panels (**a**,**b**,**e**,**f**,**i**,**j**,**m**,**n**) represent the R1 growth stage; panels (**c**,**d**,**g**,**h**,**k**,**l**,**o**,**p**) represent the R6 growth stage. Panels (**a**,**c**,**e**,**g**,**i**,**k**,**m**,**o**) represent Liaodou 14 (L14); panels (**b**,**d**,**f**,**h**,**j**,**l**,**n**,**p**) represent Liaodou 10 (L10).

A three-way ANOVA revealed that cultivar, nitrogen level, and soil extract treatment had varying degrees of influence on the expression levels of metabolism-related genes in soybean leaves and fine roots, with significant main effects observed for most genes and significant three-way interactions for the majority of indicators (Table S4). Expression analysis revealed that the CC treatment significantly downregulated the expression of *GmNRT1.1*, *GmNRT2.5*, *GmINR1*, *GmINR2*, *GmNIR*, *GmGS1*, *GmGDH1*, and *GmFd-GOGAT* genes in leaves and fine roots, with the exception of *GmNADH-GOGAT* (Figure 2 and Table S5). At the R1 and R6 growth stages, the expression levels of *GmNRT1.1*, *GmNRT2.5*, *GmINR2*, *GmINR1*, and *GmNIR* in L10 under the CC treatment were lower than those in L14. The expression of *GmGS1* in leaves was maintained at higher levels in the tolerant cultivar, with L14 showing 2.4-fold higher expression than L10 under the medium-nitrogen (MN) treatment at the R6 growth stage (Figure 2 and Table S5). The expression of *GmGDH1* decreased under the CC treatment (Table S5), contrasting with the trend observed for GDH activity, while the expression of *GmNADH-GOGAT* increased under the CC treatment (Figure 2 and Table S5).

### 2.3. Integrated Analysis of Nitrogen Metabolism Indicators

**Figure 3:**
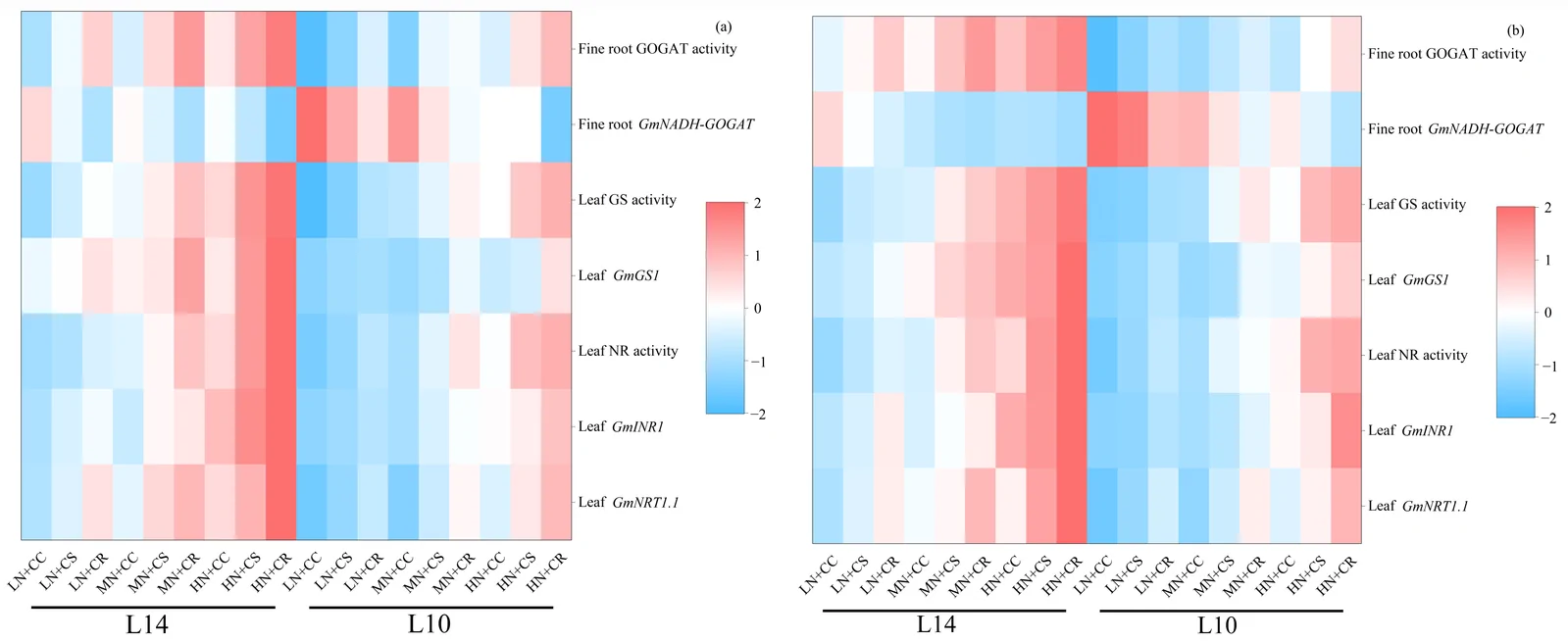
Heatmaps of nitrogen metabolism enzyme activities and relative expression levels of key nitrogen metabolism-related genes in soybean under varying nitrogen levels. Rows represent individual indicators; Columns represent treatment combinations. Data are normalized by Z-score across rows. Colors indicate relative expression levels: red, high; blue, low. Abbreviations: L14, Liaodou 14; L10, Liaodou 10; LN, low nitrogen; MN, medium nitrogen; HN, high nitrogen; CC, soybean continuous cropping soil extract; CS, soy-bean continuous cropping soil extract + sodium orthovanadate (Na_3_VO_4_); CR, soybean crop rotation soil extract. Panel (**a**) represents the initial flowering growth stage (R1); panel (**b**) represents the full seed growth stage (R6).

To visualize the overall response patterns of nitrogen metabolism indicators to continuous cropping stress and nitrogen supply, heatmaps were constructed using key enzyme activities and relative expression levels of nitrogen metabolism at the R1 (Figure 3a) and R6 growth stages (Figure 3b), facilitating comparison between gene expression and corresponding enzyme activities. The CC treatment reduced GOGAT activity in fine roots and NR and GS activities, in addition to *GmNRT1.1*, *GmINR1*, and *GmGS1* expression in leaves compared with the CR treatment, as reflected by the predominance of blue colors in the CC treatment versus red colors in the CR treatment. This pattern was consistent across both cultivars but was more pronounced in L10. The HN treatment partially alleviated the inhibitory effects of the CC treatment, shifting the color from blue to red, particularly in L14. Under CC, the LN treatment resulted in the lowest NR and GS activities and *GmNRT1.1*, *GmINR1*, and *GmGS1* expression in leaves, in addition to the lowest GOGAT activity in fine roots. Most gene expression levels showed consistent trends with their corresponding enzyme activities across treatments; notably, the expression of *GmNADH-GOGAT* in fine roots exhibited a trend opposite to that of GOGAT activity.

### 2.4. Nodule Number and Nitrogenase Activity

**Figure 4:**
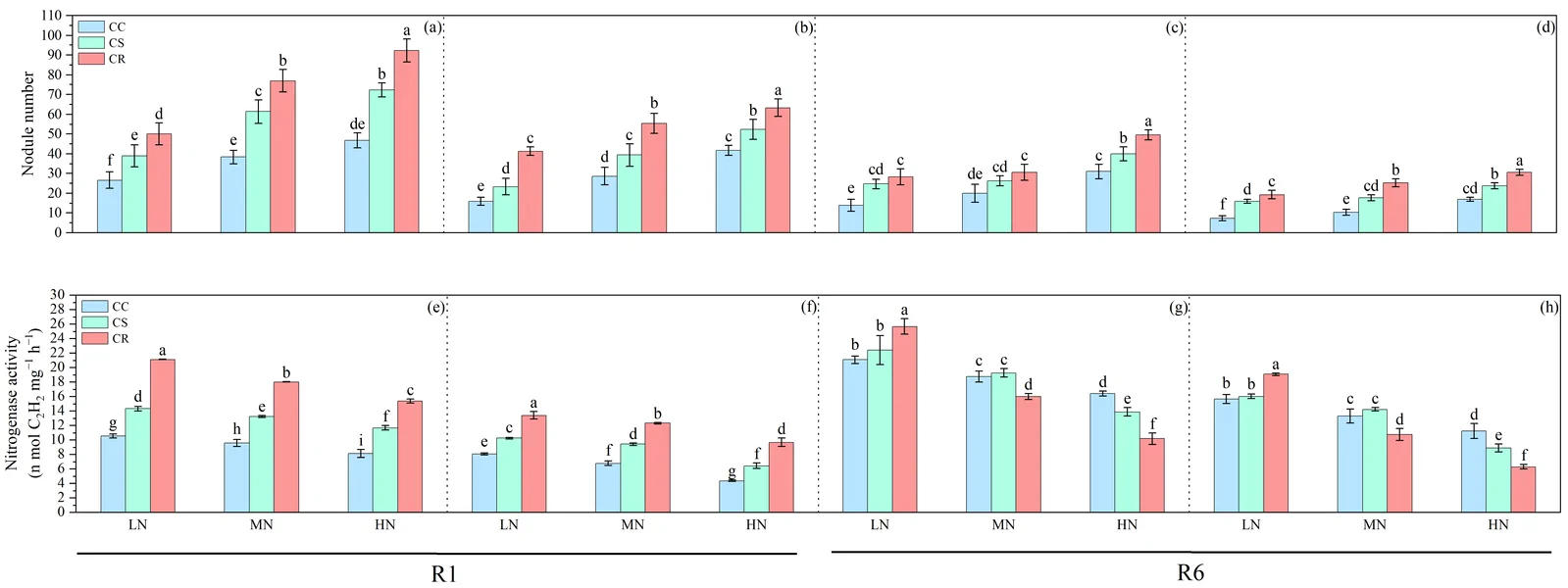
Effects of nitrogen levels on nodule number and nitrogenase activity of soybean cultivars with different continuous cropping tolerance in 2020. Different lowercase letters above bars indicate significant differences among treatments within each cultivar at *p* < 0.05. Abbreviations: R1, initial flowering growth stage; R6, full seed growth stage; LN, low nitrogen; MN, medium nitrogen; HN, high nitrogen; CC, soybean continuous cropping soil extract; CS, soybean continuous cropping soil extract + sodium orthovanadate (Na_3_VO_4_); CR, soybean crop rotation soil extract. Panels (**a**,**b**,**e**,**f**) represent the R1 growth stage; panels (**c**,**d**,**g**,**h**) represent the R6 growth stage. Panels (**a**,**c**,**e**,**g**) represent Liaodou 14 (L14); panels (**b**,**d**,**f**,**h**) represent Liaodou 10 (L10).

A three-way ANOVA revealed that cultivar, nitrogen level, and soil extract had highly significant effects on most nodule number and nitrogenase activity parameters across the two growing seasons (2020 and 2021), with significant cultivar × soil extract interactions (Table S1). At the R1 growth stage under the CC treatment, L14 had 55.96 and 58.11 nodules per plant in 2020 and 2021, respectively, whereas L10 had only 40.15 and 39.19 nodules per plant, accounting for 71.74% and 67.43% of L14, respectively. At the R6 growth stage, there was a greater difference between cultivars, with the nodule number in L10 decreasing to 62.22% and 65.66% of that in L14 for the two growing seasons, respectively. Nitrogenase activity peaked at the R6 growth stage, and L14 showed significantly higher nitrogenase activity than L10 under the CC treatment (Figure 4 and Figure S1). Nitrogen level significantly affected nodule number and nitrogenase activity. The nodule number was the highest under the HN treatment, whereas nitrogenase activity remained relatively low; under the LN treatment, the nodule number decreased while nitrogenase activity reached the highest level. All nodule numbers and most levels of nitrogenase activity under the CS treatment were higher than those under the CC treatment (Figure 4 and Figure S1). Two-year data showed that nodule number and nitrogenase activity in 2021 were slightly higher than in 2020 across all treatments, whereas the relative trends among treatments were consistent (Figure 4 and Figure S1).

### 2.5. Nitrogen Accumulation and Distribution

#### 2.5.1. Nitrogen Content

**Figure 5:**
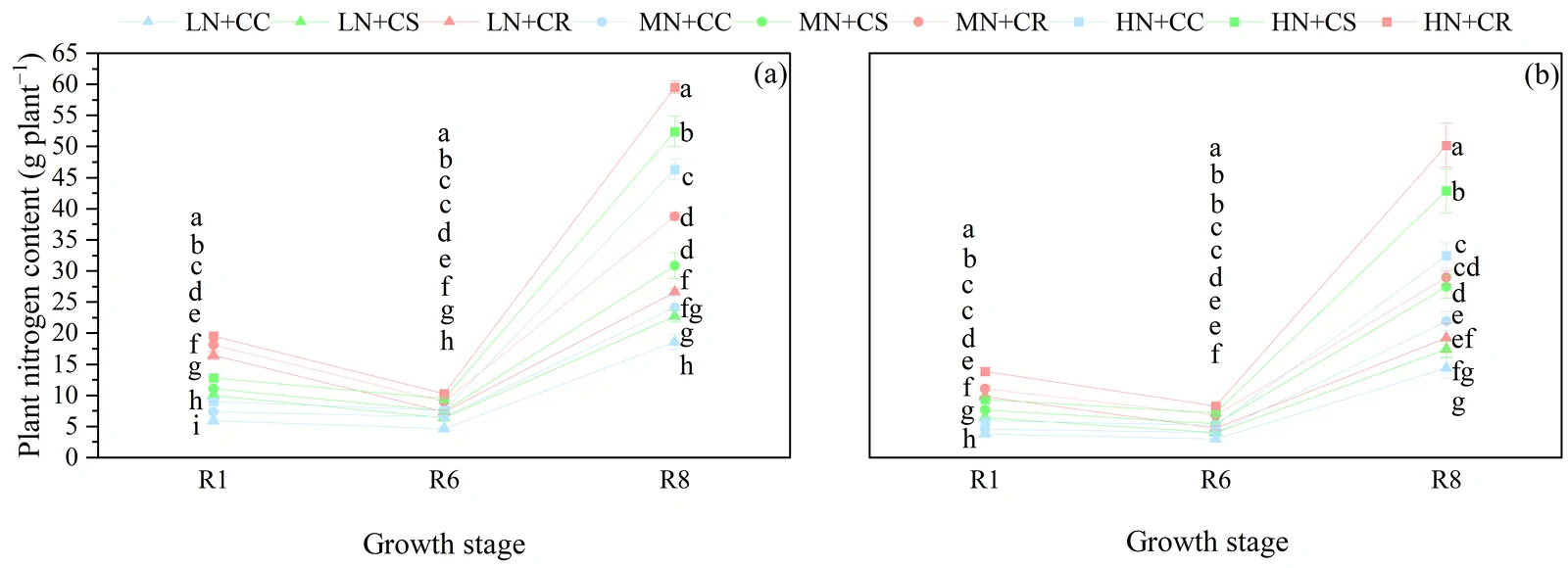
Dynamic changes in total nitrogen accumulation at different growth stages of soybean cultivars differing in continuous cropping tolerance under varying nitrogen levels in 2020. Different lowercase letters in the figure indicate significant differences among treatments at the same growth stage at *p* < 0.05. Abbreviations: R1, initial flowering growth stage; R6, full seed growth stage; R8, full maturity growth stage; LN, low nitrogen; MN, medium nitrogen; HN, high nitrogen; CC, soybean continuous cropping soil extract; CS, soybean continuous cropping soil extract + sodium orthovanadate (Na_3_VO_4_); CR, soybean crop rotation soil extract. Panel (**a**) represents Liaodou 14 (L14); panel (**b**) represents Liaodou 10 (L10).

A three-way ANOVA revealed that cultivar, nitrogen level, and soil extract treatment had varying degrees of influence on shoot, root, and total nitrogen contents, with significant main effects observed for most nitrogen content indicators and significant three-way interactions for most measured variables across the two growing seasons (2020 and 2021) (Table S2). Shoot, root, and total nitrogen contents were significantly inhibited by continuous cropping at the R1, R6, and R8 growth stages. Furthermore, L14 exhibited a higher nitrogen content than L10 across all growth stages. (Figure 5 and Figure S2 and Table S7). Shoot and root nitrogen contents differed significantly among nitrogen levels, with the highest values observed under the HN treatment. Both shoot and root nitrogen contents under the CS treatment were higher than those under the CC treatment. From the R1 to R8 growth stage, the nitrogen content initially decreased and then increased, reaching the lowest value at the R6 growth stage, with a smaller increase observed under the CC treatment than the CR treatment. This trend was consistent across observations in 2020 and 2021 (Figure 5 and Figure S2 and Table S7).

#### 2.5.2. Nitrogen Distribution Ratio

**Figure 6:**
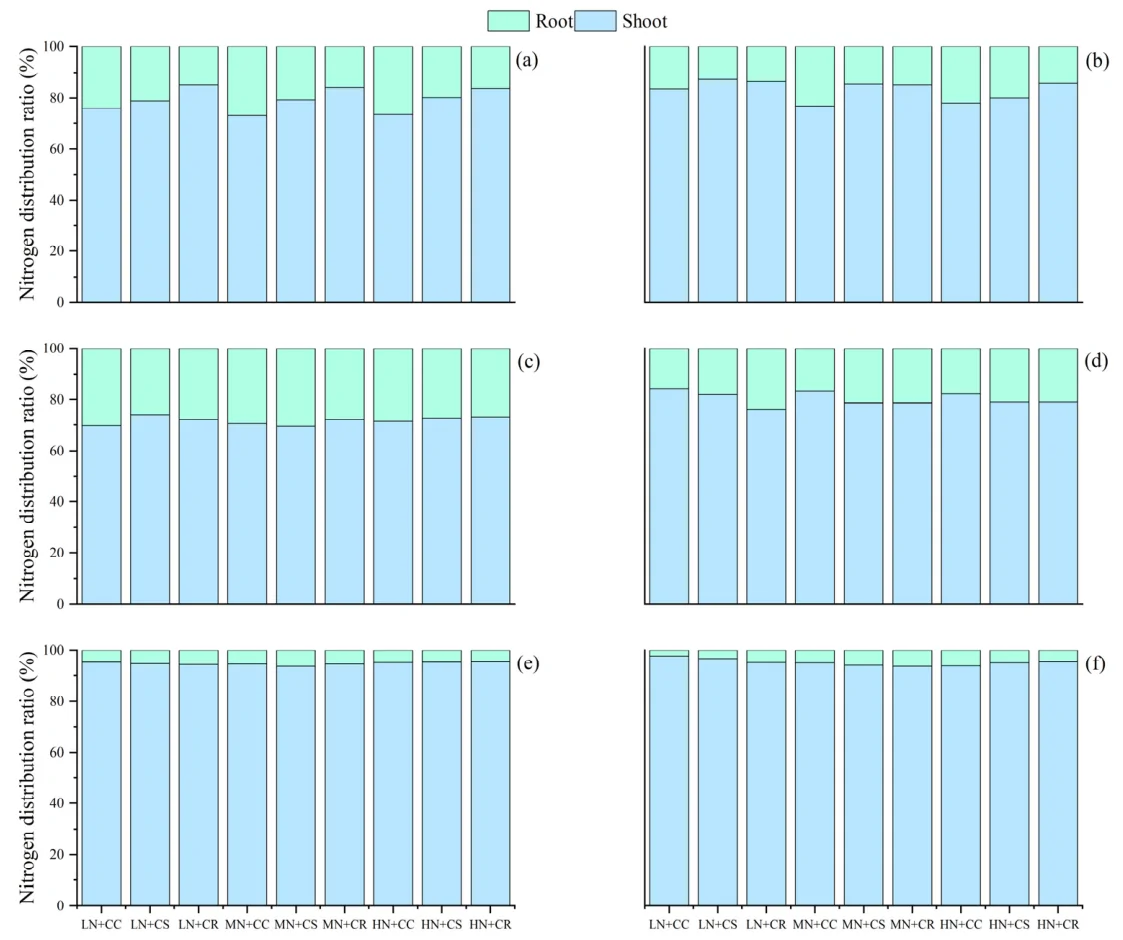
Nitrogen distribution ratio between shoots and roots of soybean cultivars differing in continuous cropping tolerance under varying nitrogen levels in 2020. Abbreviations: LN, low nitrogen; MN, medium nitrogen; HN, high nitrogen; CC, soybean continuous cropping soil extract; CS, soybean continuous cropping soil extract + sodium orthovanadate (Na_3_VO_4_); CR, soybean crop rotation soil extract. Panels (**a**) and (**b**) represent the initial flowering growth stage (R1); panels (**c**) and (**d**) represent the full seed growth stage (R6); panels (**e**) and (**f**) represent the full maturity growth stage (R8). Panels (**a**,**c**,**e**) represent Liaodou 14 (L14); panels (**b**,**d**,**f**) represent Liaodou 10 (L10).

The CC treatment not only reduced the nitrogen content but also significantly altered the distribution of nitrogen between shoots and roots across the two growing seasons (2020 and 2021) (Figure 6 and Figure S3). The shoot nitrogen distribution ratios of L14 and L10 initially increased and then decreased from the R1 to R8 growth stage in both 2020 and 2021. At the R1 growth stage, the shoot nitrogen distribution ratios under the CC treatment were higher than those under the CR treatment for the two-year observations. At the R6 growth stage, L14 exhibited a higher shoot nitrogen distribution ratio under the CC treatment than under the CR treatment, whereas the opposite pattern was observed for L10 in both years. The shoot nitrogen distribution ratio under the CS treatment was intermediate between the CC and CR treatments (Figure 6 and Figure S3).

## 3. Discussion

Nitrogen is a key limiting factor for soybean yield, and nitrogen fertilizer application can promote root growth and nitrogen accumulation, which is critical for improving soybean yield [19,20,21]. Continuous cropping of soybean results in soil nutrient depletion, which requires timely nutrient supplementation to satisfy the requirements of soybean growth and development. Nitrogen fertilization can increase crop yield [22] and alleviate continuous cropping obstacles. Nevertheless, the physiological and biochemical mechanisms underlying the responses of continuous-cropping-tolerant soybean cultivars to different nitrogen levels remain poorly documented. The present study evaluated the effects of different nitrogen levels on nitrogen metabolism enzyme activities, gene expression, nodulation and nitrogen fixation, and nitrogen accumulation in continuously cropped soybean.

### 3.1. Differential Regulation of Nitrogen Metabolism Enzyme Activities by Nitrogen Levels Among Cultivars

Nitrogen metabolism enzyme activity is a vital physiological index reflecting a plant’s nitrogen assimilation capacity under abiotic stress [4]. The results of this study demonstrate that the soybean continuous cropping soil extract (CC) treatment significantly inhibited the activities of key nitrogen metabolism enzymes in soybean leaves and fine roots; however, this inhibition was markedly alleviated by the high-nitrogen (HN) treatment, with the continuous-cropping-tolerant cultivar Liaodou 14 (L14) exhibiting significantly less inhibition than the continuous-cropping-sensitive cultivar Liaodou 10 (L10) across all nitrogen levels (Figure 1 and Table S6). This finding is consistent with the results of our previous study [23], in which cultivar improvement significantly increased nitrogen content and accumulation in soybean under continuous cropping. As the rate-limiting enzyme in nitrate assimilation, nitrate reductase (NR) activity was significantly reduced under the CC treatment, while the HN treatment partially restored NR activity, with a more pronounced recovery effect observed in L14. Notably, glutamate dehydrogenase (GDH) activity exhibited an increasing trend under the CC treatment, whereas glutamine synthetase (GS) and glutamate synthase (GOGAT) activities were inhibited. Previous studies have revealed that when the GS-GOGAT cycle was inhibited, GDH activity increased by 0.4- to 1.9-fold to maintain nitrogen assimilation. These published findings lend plausibility to our observation that the GDH pathway could function as a compensatory route to sustain nitrogen assimilation under continuous cropping stress [24]. Under our experimental conditions, the enzyme activities under the soybean continuous cropping soil extract + sodium orthovanadate (CS) treatment were intermediate between those of the CC treatment and the soybean crop rotation soil extract (CR) treatment. It should be noted that the working concentration of sodium orthovanadate used in this study (100 µM) was selected based on concentration ranges commonly adopted in plant physiological studies [17,25,26]. A 200 mM stock solution was prepared and diluted to the working concentration of 100 µM prior to use. At this concentration, soybean plants grew normally throughout the experimental period without visible toxic symptoms, indicating that this concentration is suitable for long-term treatment of intact soybean plants.

### 3.2. Differential Regulation of Nitrogen Metabolism-Related Gene Expression by Varying Nitrogen Levels Among Cultivars

Transcriptional reprogramming of nitrogen metabolism-related genes may constitute one core molecular adaptive strategy for soybean under stress, and such transcriptional responses are strongly modulated by external nitrogen supply; adequate nitrogen availability can partially relieve the transcriptional repression triggered by continuous cropping [27,28]. To further elucidate the molecular mechanisms by which continuous cropping and nitrogen levels regulate nitrogen metabolism in soybean, the present study analyzed the expression patterns of nitrogen metabolism-related genes. The CC treatment significantly downregulated the expression of nitrogen metabolism-related genes, and nitrogen levels played an important regulatory role in this process; specifically, the HN treatment mitigated the transcriptional repression induced by continuous cropping (Figure 2 and Table S5). The expression levels of *GmNRT1.1* and *GmNRT2.5*, encoding nitrate transporter proteins, were significantly induced by the HN treatment, while they were significantly suppressed by the CC treatment. The downregulation of *GmINR1* and *GmINR2* expression corresponded with reduced NR activity, and high nitrogen supply effectively restored their transcription levels. These parallel changes raise the possibility that continuous-cropping-mediated inhibition of NR activity may at least partly occur at the transcriptional level, and that nitrogen supply could relieve such transcriptional suppression [27,28]. Under the CC treatment, *GmGDH1* expression decreased, contrary to the increasing trend observed for GDH enzyme activity (Figure 1). Similarly, *GmNADH-GOGAT* also showed the opposite trend to GOGAT enzyme activity under the CC treatment. This uncoupling between gene transcription and enzyme activity suggests that post-transcriptional regulation may play a role in modulating key nitrogen metabolism enzymes under continuous cropping stress. However, multiple mechanisms may explain this phenomenon, including miRNA-mediated mRNA degradation, translational repression, and protein phosphorylation. Future studies need to verify protein abundance through Western blotting or enzyme-kinetic analysis to confirm these possibilities. These studies will contribute to a deeper understanding of the molecular mechanisms by which continuous cropping stress regulates nitrogen metabolism at multiple levels. Across all nitrogen level treatments, L14 exhibited consistently higher gene expression levels than the sensitive cultivar [29], suggesting that L14 may possess more robust transcriptional regulatory capacity for nitrogen metabolism genes under varying nitrogen and continuous cropping conditions [27,28]. Across different nitrogen levels, L14 generally exhibited higher transcript levels for most detected nitrogen metabolism-related genes than L10, except *GmNADH-GOGAT*, which showed lower expression in L14 [29]. This finding reflects that the continuous-cropping-tolerant cultivar generally possesses stronger transcriptional regulatory capacity for nitrogen metabolism-related genes under combined continuous cropping stress and variable nitrogen level conditions.

### 3.3. Differential Regulation of Nodule Number and Nitrogenase Activity by Varying Nitrogen Levels Among Cultivars

Root nodulation and nitrogen fixation capacity constitute crucial symbiotic nitrogen acquisition strategies for soybean; symbiotic traits, including nodule number and nitrogenase activity, are jointly modulated by continuous cropping stress and external nitrogen availability [30,31]; however, the mechanisms underlying their co-regulation remain poorly understood. Nodule number and nitrogenase activity were significantly affected by continuous cropping stress and nitrogen levels (Figure 4). The CC treatment inhibited the nodule number significantly more in L10 than in L14. Previous studies reported that nitrogen fertilization reduces nodule nitrogenase activity and suppresses symbiotic nitrogen fixation performance [32]. Consistent with these reports, nitrogenase activity decreased with rising nitrogen levels in the present study. This result is fully consistent with the classical view that nitrate inhibits nodule nitrogen fixation function. Additionally, nodule number increased with rising nitrogen contents in the present study, possibly because under low-nitrogen conditions, soybean plants suffered severe continuous cropping stress, and the CC treatment exerted strong inhibitory effects on nodule formation, resulting in fewer nodules. Supplemental high nitrogen supply improved plant nitrogen nutrition status and alleviated plant damage induced by continuous cropping stress, weakening the stress-derived inhibition of nodule formation and consequently leading to more nodules under the HN treatment. Moreover, both nodule number and nitrogenase activity were significantly higher in L14 than in L10, indicating that the continuous-cropping-tolerant cultivar can establish more nodules and mitigate the generation of ineffective nodules. Under continuous cropping conditions, the symbiotic association between soybean and rhizobia is weakened, while daidzein secreted by roots can sustain the population of specific rhizobial strains [33]. Nodule number was consistently higher under the CS treatment than the CC treatment, while nitrogenase activity showed this trend in most instances, reflecting differences triggered by sodium orthovanadate addition. It is worth noting that the HN treatment improved plant nitrogen metabolism enzyme activities, gene expression, and nitrogen accumulation under continuous cropping conditions. However, these beneficial changes did not extend to symbiotic nodule function, which remained impaired under continuous cropping stress. Accordingly, our results support the classical view that high mineral nitrogen inhibits nodule physiological performance, which is consistent with previous reports [32].

### 3.4. Differential Regulation of Nitrogen Accumulation and Distribution by Nitrogen Levels Among Cultivars

Symbiotic nitrogen fixation capacity and nitrogen metabolism enzyme activities jointly govern plant nitrogen acquisition, which is ultimately reflected in nitrogen accumulation and distribution within soybean [3,34,35]. Accordingly, this study further investigated the differences in nitrogen content and distribution among various treatments. The CC treatment significantly reduced the nitrogen content in soybean plants, with L14 maintaining higher levels than L10 across all growth stages, and the HN treatment partially compensated for the reduction in nitrogen accumulation caused by the CC treatment (Figure 5 and Table S7). Our previous work also confirmed that cultivar improvement can optimize nitrogen accumulation in continuously cropped soybean [23]. The nitrogen content initially decreased from the R1 growth stage to the R6 growth stage and then increased from R6 to R8, with the lowest value observed at R6, which is attributable to nitrogen dilution effects and remobilization processes during soybean development. The magnitude of change in nitrogen content under continuous cropping was smaller than that under rotational cropping, indicating that continuous cropping inhibited nitrogen uptake and accumulation by soybean. The CC treatment also significantly altered the distribution of nitrogen between shoots and roots (Figure 6). Under the CC treatment, a general trend for elevated proportional nitrogen allocation to roots points to constraints on root-to-shoot nitrogen translocation imposed by continuous cropping stress [36]. Likewise, L14 displays a general trend of higher root nitrogen allocation ratios over growth stages, which could plausibly represent a nitrogen allocation strategy contributing to cultivar adaptation to continuous cropping stress [36,37].

### 3.5. The Capacity to Maintain Nitrogen Metabolic Function of Continuous-Cropping-Tolerant Soybean Cultivar

Our previous studies have shown that the continuous-cropping-tolerant soybean cultivar L14 can alleviate continuous cropping obstacles by recruiting more beneficial bacteria, altering the structure and function of microbial communities, and improving soil nutrient cycling [29]. Integrating the results of enzyme activities, gene expression, nodulation and nitrogen fixation, and nitrogen accumulation, the present study further suggests that the continuous-cropping-tolerant cultivar L14 may exhibit a stronger capacity to maintain nitrogen metabolic function under continuous cropping stress; specifically, it may maintain coordinated activities of core nitrogen metabolism enzymes and transcript abundance of relevant functional genes, suffer less damage in the nodule fixation apparatus, and achieve enhanced nitrogen acquisition and allocation capacity. Under the present study’s experimental conditions, appropriate nitrogen supply may achieve optimal balance and coordinate nitrogen metabolism and symbiotic nitrogen fixation, though this physiological inference requires further verification under diverse growing conditions. It should be noted that no rhizobium inoculation was performed in this experiment, which may limit the absolute nodule formation capacity. This methodological choice was based on our research objective, which focuses on the effects of continuous cropping stress on soybean nitrogen metabolism. Accordingly, data related to nodulation and nitrogen fixation should mainly be interpreted based on relative trends across treatments. In addition, although most indicators under the CS treatment showed intermediate values between CC and CR, sodium orthovanadate is a broad-spectrum inhibitor of phosphatases and H^+^-ATPase. Such observations cannot be uniquely attributed to the suppression of specific transport processes and may also reflect its direct interference with physiological processes such as nitrogen metabolism. Further verification is required in future studies. Collectively, these results raise the possibility that exploiting genotypic differences in the capacity to maintain nitrogen metabolic function among soybean cultivars, combined with appropriate nitrogen application strategies, could help mitigate the adverse effects of continuous cropping stress on soybean performance.

## 4. Materials and Methods

### 4.1. Experimental Design

The sand culture pot experiment was conducted at the experimental station of the College of Agronomy, Shenyang Agricultural University, Shenyang, Liaoning Province, in 2020 and 2021. Two soybean cultivars with contrasting continuous cropping tolerances were used as experimental materials: Liaodou 14 (continuous-cropping-tolerant) and Liaodou 10 (continuous-cropping-sensitive). Quartz sand was used as the growth substrate in hard plastic pots (25 cm in height and 15 cm in diameter at both the top and bottom), with each pot filled with 10 kg of quartz sand, and no exogenous rhizobia were inoculated.

Three nitrogen levels were established in this experiment: low-nitrogen treatment [LN, 0.2 mM Ca(NO_3_)_2_, 0.009 μM (NH_4_)_6_Mo_7_O_24_, 1.8 mM CaCl_2_], medium-nitrogen treatment [MN, 2 mM Ca(NO_3_)_2_, 0.09 μM (NH_4_)_6_Mo_7_O_24_], and high-nitrogen treatment [HN, 2 mM Ca(NO_3_)_2_, 0.09 μM (NH_4_)_6_Mo_7_O_24_, 36 mM NaNO_3_, 0.81 μM (NH_4_)_2_SO_4_]. Except for nitrogen-regulating components, all other nutrient elements were supplied according to the formula of half-strength Hoagland hydroponic nutrient solution. Soil extract treatments were applied at the first trifoliate leaf growth stage (V1) of soybean seedlings, including three groups: 1 g·L^−1^ of soybean continuous cropping soil extract (CC), 1 g·L^−1^ of soybean continuous cropping soil extract + sodium orthovanadate (final concentration of 100 μM, prepared from a 200 mM stock solution) [17,25,26] (CS), and 1 g·L^−1^ of soybean crop rotation soil extract (CR). Each treatment contained six replicates, with three seedlings retained per pot. Sampling was conducted at the initial flowering growth stage (R1) of soybean in both years, with differences in measured indicators. All experimental indicators were determined in 2020, while only nitrogen content, nitrogenase activity, and nodule number were measured in 2021 to verify the annual repeatability of key indicators.

Uniform nutrient solution replenishment was adopted for daily cultivation management, and the nutrient solution was replenished every two days. Each pot was irrigated with 250 mL of nutrient solution every two days, with approximately 39 irrigations over the 78-day growth period. The total nitrogen supplied per pot across the whole experiment was 54.6 mg for LN, 546.0 mg for MN, and 5460.0 mg for HN. These nitrogen treatment gradients were designed following the strategy reported in published soybean sand culture studies [38,39], setting low-nitrogen, medium-nitrogen, and high-nitrogen treatments to induce distinct physiological responses in soybean. During cultivation, the concentrations of macroelements in the nutrient solution were adjusted appropriately according to different growth stages of soybean.

### 4.2. Determination Items and Methods

In the sand culture experiment, each treatment was replicated 6 times. The leaves and roots from 3 replicates were used to determine nitrogen metabolism enzymes and nitrogen metabolism-related genes in leaves and roots, while the shoots from the other 3 replicates were used to determine nitrogen content, and the roots were also used to determine nodule number, nitrogenase activity, and nitrogen content.

#### 4.2.1. Nodule Number and Nitrogenase Activity

Soybean roots were collected at the R1 and full seed growth stages (R6). Nitrogenase activity was determined using an Agilent 7890A gas chromatograph (Agilent Technologies, Santa Clara, CA, USA) through the acetylene reduction method. The cleaned soybean roots were placed in sealed vials with rubber stoppers. First, 25 mL of air was withdrawn from the vial using a syringe, and then 25 mL of acetylene gas was injected into the vial. After incubation at room temperature for 1 h, 1 mL of the mixed gas was immediately extracted from the vial using a micro-syringe and injected into the gas chromatograph to determine ethylene content. Following the gas measurement, the roots were removed from the vials, and the nodule number was recorded. The gas chromatograph was equipped with a 13 m stainless steel packed column. A flame ionization detector (FID) was used at a temperature of 250 °C, with a column temperature of 60 °C and an injector temperature of 120 °C. N_2_ was used as the carrier gas, with flow rates of 35 mL min^−1^ for N_2_, 50 mL min^−1^ for H_2_, and 350 mL min^−1^ for air. The amount of reduced acetylene was calculated as follows:

Ethylene content (%) = Standard ethylene gas content (%) × Sample ethylene gas peak height (mm)/Standard ethylene gas peak height (mm) × 100;

Pure ethylene volume (L) = Ethylene content (%) × Gas phase volume (L);

Ethylene formation (n mol) = Pure ethylene gas volume (L)/22.4 (L) × 10^9^.

The ethylene formation represents the amount of reduced acetylene, and nitrogenase activity is expressed as nmol C_2_H_2_ mg^−1^ h^−1^ [40].

#### 4.2.2. Nitrogen Content

Soybean shoots and roots were collected at the R1, R6, and full maturity growth stages (R8). The shoots and roots were oven-dried at 105 °C for 30 min to deactivate enzymes, and then dried at 80 °C to a constant weight, ground into powder, and aliquoted. The samples were digested using the H_2_SO_4_-H_2_O_2_ digestion method, and nitrogen content was determined using an automated Kjeldahl apparatus (KJELTEC™ 8400; FOSS Analytical, Hillerød, Denmark).

#### 4.2.3. Nitrogen Metabolism Enzymes

Soybean leaves and fine roots (defined as lateral roots of the second order or higher, rinsed with water) were collected at the R1 and R6 growth stages to determine nitrogen metabolism enzymes. Leaves and fine roots (defined as lateral roots of the second order or higher, rinsed with water) were collected at the R1 and R6 growth stages. The activities of nitrate reductase (NR) and glutamine synthetase (GS) were determined according to Xu et al. [41]. Nitrite reductase (NiR) activity was assayed following Rajasekhar et al. [42], and the activities of glutamate dehydrogenase (GDH) and glutamate synthase (GOGAT) were measured following Lin et al. [43].

#### 4.2.4. Nitrogen Metabolism-Related Genes

Soybean leaves and fine roots were collected at the R1 and R6 growth stages. Total RNA was extracted from leaves and fine roots using a plant total RNA isolation kit (Qiagen, Hilden, Germany), and cDNA was synthesized using the SuperScript III First-Strand Synthesis System (Thermo Fisher Scientific, Waltham, MA, USA) according to the manufacturer’s instructions [44]. RNA purity and concentration were determined using a NanoDrop UV spectrophotometer (Thermo Fisher Scientific, Waltham, MA, USA). All primers were validated by analyzing product size and nucleotide sequence, and the primer sequences are shown in Table 1. RT-qPCR analysis was performed using a 15 μL reaction system [44], consisting of 7.5 μL of SYBR qPCR mix (Vazyme, Nanjing, China), 1.2 μL of PCR forward primer (10 μM), 1.2 μL of PCR reverse primer (10 μM), 3 μL of DNA (100 ng), and 2.1 μL of ddH_2_O.

### 4.3. Statistical Analysis

All measured data were processed using Microsoft Excel 2010. Statistical analyses were performed with SPSS software (Version 22.0, SPSS Inc., Chicago, IL, USA), and means were compared using Duncan’s new multiple range test at the significance level of *p* < 0.05. Graphs were generated with OriginPro 2021 (OriginLab Corporation, Northampton, MA, USA). Given that different sets of indicators were measured in the two experimental years, statistical analyses were performed separately for the data obtained in 2020 and 2021.

## 5. Conclusions

This study reveals regulatory mechanisms underlying nitrogen metabolic and nodulation symbiotic nitrogen fixation responses of soybean cultivars with contrasting continuous cropping tolerances under varying stress levels. The results indicate that the CC treatment severely inhibited nitrogen metabolism and symbiotic nitrogen fixation processes in soybean. In contrast, L14 exhibited a stronger capacity to maintain nitrogen metabolic function, which enabled stable enzyme performance, sustained gene expression, and efficient nitrogen translocation and allocation under the CC treatment. This may constitute a plausible adaptive mechanism for continuous-cropping-tolerant soybean to cope with continuous cropping stress and maintain high nitrogen use efficiency. Nitrogen levels exerted differential regulatory effects on soybean adaptation to continuous cropping. Under the present study’s experimental conditions, appropriate nitrogen supply that coordinates plant metabolism and symbiotic nitrogen fixation provides a theoretical reference for alleviating soybean continuous cropping obstacles, though further field trials are required to verify its practical application. The intermediate physiological responses triggered by sodium orthovanadate further indicated its potential to alleviate continuous cropping obstacles in soybean. Collectively, this study suggests that a stronger capacity to maintain nitrogen metabolic function could represent an important adaptive feature of continuous-cropping-tolerant soybean cultivars in coping with continuous cropping stress. It also provides an important theoretical basis and references for dissecting continuous cropping tolerance mechanisms and precise nitrogen cultivation regulation of soybean in continuous cropping agroecosystems.

## Figures and Tables

**Table 1:** Soybean gene locus, Arabidopsis homologues and q RT-PCR primer sequences.

Gene Name	Primer (5′–3′)	Soybean Locus	Arabidopsis Locus	Amino Acid Identity
*GmNRT1.1*	Forward ACACAATGCAGTGGTTCCCA	Glyma.17G139400.1	AT1G12110.1	71%
	Reverse GTACGTAAATGCCTCCCCCG			
*GmNRT2.5*	Forward AGGTCCACGTTATGGCTGTG	Glyma.11G195200.1	AT1G12940.1	69.40%
	Reverse GCCAAACAATCTGGCTGCAA			
*GmINR1*	Forward GATCCTCGCCCGTATGAAGG	Glyma.06G109200.1	AT1G37130.1	82.90%
	Reverse CATACTTGGACCCACCACCC			
*GmINR2*	Forward CTCGCCTACATGCAAAACGG	Glyma.13G084000.1	AT1G37130.1	78.80%
	Reverse GCGCTTCAACCATTTGACCA			
*GmNIR*	Forward AACCCCGCCATGTCAAACTT	Glyma.02G132100.1	AT2G15620.1	84.50%
	Reverse TTGCAGGCATGTAAGCCAGA			
*GmGS1*	Forward TAGAGGGTGACTGGAATGGAGCAG	Glyma.11G215500.1	AT1G66200.3	90.40%
	Reverse TGATGTGATCCTTGTGGCGTAGC			
*GmGDH1*	Forward TGATGGCACCTTGCAATCTT	Glyma.16G041200.1	AT3G03910.1	94.40%
	Reverse TTCCTCCTTTCATGGGGCCT			
*GmFd-GOGAT*	Forward TAGCACTCACCAGTTTGCCT	Glyma.03G128300.1	AT2G41220.1	87.60%
	Reverse CCGACATGTCTCCAATGCCA			
*GmNADH-GOGAT*	Forward AGTGACAAACCCACCGATTGA	Glyma.06G127400.1	AT5G53460.1	86.50%
	Reverse AGGAAAGACGGTGGCATTGA			

## Data Availability

The original contributions presented in this study are included in the article/Supplementary Materials. Further inquiries can be directed to the corresponding author.

## Supplementary Materials

The following supporting information can be downloaded at: https://www.mdpi.com/article/10.3390/plants15182839/s1, Table S1: Three-way ANOVA of nitrogen level, soil leaching liquor, and cultivar on nitrogenase activity and nodule number per plant in soybean; Table S2: Three-way ANOVA of nitrogen level, soil leaching liquor, and cultivar on N content in soybean; Table S3: Three-way ANOVA of nitrogen level, soil leaching liquor, and cultivar on nitrogen-metabolizing enzyme activities in soybean; Table S4: Three-way ANOVA of nitrogen level, soil leaching liquor, and cultivar on expression of nitrogen-metabolism-related genes in soybean; Table S5: Expression levels of nitrogen metabolism-related genes in leaves and fine roots of soybean under different treatments; Table S6: Activities of nitrogen metabolism enzymes in leaves and fine roots of soybean under different treatments; Table S7: Nitrogen content in shoots and fine roots of soybean under different treatments. Figure S1: Effects of nitrogen levels on nodule number and nitrogenase activity of soybean cultivars with different continuous cropping tolerance in 2021. Figure S2. Dynamic changes in total nitrogen accumulation at different growth stages of soybean cultivars differing in continuous cropping tolerance under varying nitrogen levels in 2021. Figure S3. Nitrogen distribution ratio between shoots and roots of soybean cultivars differing in continuous cropping tolerance under varying nitrogen levels in 2021.
