# Supplementary material for: Continuous Cropping Differentially Regulates Nitrogen Metabolism and Nodulation in Soybean Cultivars with Contrasting Continuous Cropping Tolerance Under Varied Nitrogen Levels
Source: Plants (Basel). 2026 Sep 17;15(18):2839. doi: 10.3390/plants15182839 (PMC13610383; doi:10.3390/plants15182839)
Supplement: Supplementary file 1 [file plants-15-02839-s001.zip › plants-4544237-supplementary-update.pdf]

Supplementary Table S1 Three-way ANOVA of nitrogen level, soil leaching liquor, and cultivar on nitrogenase activity and nodule number per plant in soybean.

**Year**	**Growth stage**	**Trait**	**Nitrogen level(N)**	**Soil leaching liquor(S)**	**Cultivar(C)**	**N×S**	**N×C**	**S×C**	**N×S×C**
2020	R1	Nitrogenase activity	**<0.001**	**<0.001**	**<0.001**	**<0.001**	**<0.001**	**<0.001**	**<0.001**
		Nodule number	**0.004**	**<0.001**	0.44	**<0.001**	**<0.001**	**<0.001**	**<0.001**
	R6	Nitrogenase activity	**<0.001**	**<0.001**	**0.049**	**<0.001**	**<0.001**	**<0.001**	**<0.001**
		Nodule number	**<0.001**	**<0.001**	**0.01**	**<0.001**	**<0.001**	**<0.001**	**<0.001**
2021	R1	Nitrogenase activity	**<0.001**	**<0.001**	**0.004**	**<0.001**	**<0.001**	**<0.001**	**<0.001**
		Nodule number	**0.001**	**<0.001**	0.12	**<0.001**	**<0.001**	**<0.001**	**<0.001**
	R6	Nitrogenase activity	**<0.001**	**<0.001**	0.31	**<0.001**	**<0.001**	**<0.001**	**<0.001**
		Nodule number	**<0.001**	**<0.001**	**0.19**	**<0.001**	**<0.001**	**<0.001**	**<0.001**

Note: Significant ($p< 0.05$) p-values are written in bold text.

Supplementary Table S2 Three-way ANOVA of nitrogen level, soil leaching liquor, and cultivar on N content in soybean.

**Year**	**Growth stage**	**Plant part**	**Nitrogen level(N)**	**Soil leaching liquor(S)**	**Cultivar(C)**	**N×S**	**N×C**	**S×C**	**N×S×C**
2020	R1	Shoot	**<0.001**	**<0.001**	**<0.001**	**<0.001**	**<0.001**	**<0.001**	**<0.001**
		Root	**<0.001**	0.05	**0.005**	**<0.001**	**<0.001**	**<0.001**	**<0.001**
		Plant	**<0.001**	**<0.001**	**<0.001**	**<0.001**	**<0.001**	**<0.001**	**<0.001**
	R6	Shoot	**0.01**	**<0.001**	0.55	**<0.001**	**<0.001**	**<0.001**	**<0.001**
		Root	**<0.001**	0.94	**0.03**	**<0.001**	**<0.001**	**<0.001**	**<0.001**
		Plant	**<0.001**	**<0.001**	0.11	**<0.001**	**<0.001**	**<0.001**	**<0.001**
	R8	Shoot	0.08	**<0.001**	**0.01**	**<0.001**	**<0.001**	**<0.001**	**<0.001**
		Root	0.09	**<0.001**	0.06	**<0.001**	**<0.001**	**<0.001**	**<0.001**
		Plant	0.06	**<0.001**	**0.03**	**<0.001**	**<0.001**	**<0.001**	**<0.001**
2021	R1	Shoot	**<0.001**	0.31	0.30	**<0.001**	**<0.001**	**<0.001**	**<0.001**
		Root	**<0.001**	0.11	**0.01**	**<0.001**	**<0.001**	**<0.001**	**<0.001**
		Plant	**<0.001**	0.37	0.11	**<0.001**	**<0.001**	**<0.001**	**<0.001**
	R6	Shoot	**0.03**	**<0.001**	0.19	**<0.001**	**<0.001**	**<0.001**	**<0.001**
		Root	**<0.001**	0.88	**0.03**	**<0.001**	**<0.001**	**<0.001**	**<0.001**
		Plant	**<0.001**	**<0.001**	**0.01**	**<0.001**	**<0.001**	**<0.001**	**<0.001**
	R8	Shoot	0.27	**<0.001**	0.22	**<0.001**	**<0.001**	**<0.001**	**<0.001**
		Root	0.11	**<0.001**	0.08	**<0.001**	**<0.001**	**<0.001**	**<0.001**
		Plant	0.23	**<0.001**	0.18	**<0.001**	**<0.001**	**<0.001**	**<0.001**

Note: Significant ($p< 0.05$) p-values are written in bold text.

Supplementary Table S3 Three-way ANOVA of nitrogen level, soil leaching liquor, and cultivar on nitrogen-metabolizing enzyme activities in soybean.

**Plant part**	**Growth stage**	**Trait**	**Nitrogen level(N)**	**Soil leaching liquor(S)**	**Cultivar(C)**	**N×S**	**N×C**	**S×C**	**N×S×C**
Leaf	R1	NR activity	0.32	**<0.001**	**0.009**	**<0.001**	**<0.001**	**<0.001**	**<0.001**
		NiR activity	**<0.001**	**<0.001**	**<0.001**	0.77	**0.014**	0.63	0.64
		GS activity	0.09	**<0.001**	0.10	**<0.001**	**<0.001**	**<0.001**	**<0.001**

		GDH activity	**<0.001**	**<0.001**	**<0.001**	**<0.001**	**<0.001**	**<0.001**	**<0.001**
		GOGAT activity	**<0.001**	**<0.001**	**<0.001**	0.71	**0.002**	0.24	0.60
		NR activity	0.77	**<0.001**	0.49	**<0.001**	**<0.001**	**<0.001**	**<0.001**
		NiR activity	**<0.001**	**<0.001**	**<0.001**	0.54	0.06	0.40	0.64
	R6	GS activity	0.24	**<0.001**	0.14	**<0.001**	**0.03**	**<0.001**	**<0.001**
		GDH activity	**<0.001**	**<0.001**	**<0.001**	**<0.001**	**<0.001**	0.09	**<0.001**
		GOGAT activity	**<0.001**	**<0.001**	**<0.001**	**0.009**	0.14	0.71	0.11
		NR activity	**<0.001**	**<0.001**	**<0.001**	0.50	0.38	**<0.001**	0.49
		NiR activity	**<0.001**	**<0.001**	**<0.001**	0.18	**<0.001**	0.15	0.93
	R1	GS activity	**<0.001**	**<0.001**	**<0.001**	**<0.001**	**0.001**	0.08	0.86
		GDH activity	**<0.001**	**<0.001**	**<0.001**	0.07	0.51	**0.037**	0.08
Root		GOGAT activity	**<0.001**	**<0.001**	0.74	**<0.001**	**<0.001**	**<0.001**	**<0.001**
		NR activity	**<0.001**	**<0.001**	**<0.001**	0.25	**0.004**	**0.045**	0.17
		NiR activity	**<0.001**	**<0.001**	**<0.001**	0.31	0.81	0.95	0.97
	R6	GS activity	**<0.001**	**<0.001**	**<0.001**	0.22	0.17	0.98	0.66
		GDH activity	**<0.001**	**<0.001**	**<0.001**	**0.009**	**<0.001**	**0.001**	**<0.001**
		GOGAT activity	**<0.001**	0.09	**<0.001**	**<0.001**	**<0.001**	**<0.001**	**<0.001**

Note: Significant ($p< 0.05$) p-values are written in bold text.

Supplementary Table S4 Three-way ANOVA of nitrogen level, soil leaching liquor, and cultivar on expression of nitrogen-metabolism-related genes in soybean.

Plant part	Growth stage	Gene name	Nitrogen level(N)	Soil leaching liquor(S)	Cultivar(C)	N×S	N×C	S×C	N×S×C
		GmNRT1.1	**<0.001**	**<0.001**	**<0.001**	**<0.001**	**<0.001**	**<0.001**	**<0.001**
		GmNRT2.5	**<0.001**	**<0.001**	**<0.001**	**<0.001**	**<0.001**	0.08	**0.013**
		GmINR1	**<0.001**	**<0.001**	**<0.001**	**<0.001**	**<0.001**	**<0.001**	**<0.001**
		GmINR2	**<0.001**	**<0.001**	**<0.001**	**<0.001**	**<0.001**	**<0.001**	**<0.001**
	R1	*GmNIR*	**<0.001**	**<0.001**	**<0.001**	**<0.001**	**<0.001**	0.05	0.31
		GmGs1	**<0.001**	0.12	**0.003**	**<0.001**	**<0.001**	**<0.001**	**<0.001**
		GmGDH1	**<0.001**	**<0.001**	**<0.001**	**<0.001**	**<0.001**	0.53	0.94
		GmFd-GOGAT	**<0.001**	**<0.001**	**<0.001**	0.09	**<0.001**	**<0.001**	**0.01**
Leaf		*GmNADH-GOGAT*	**<0.001**	**<0.001**	**<0.001**	**0.01**	**<0.001**	0.08	**0.01**
		GmNRT1.1	**<0.001**	**<0.001**	0.16	**<0.001**	**<0.001**	**<0.001**	**<0.001**
		GmNRT2.5	**<0.001**	**<0.001**	**<0.001**	**<0.001**	**<0.001**	**<0.001**	**<0.001**
		GmINR1	**<0.001**	**<0.001**	**<0.001**	**<0.001**	**<0.001**	**<0.001**	**<0.001**
		GmINR2	**<0.001**	**<0.001**	**<0.001**	**<0.001**	**<0.001**	**<0.001**	**<0.001**
	R6	*GmNIR*	**<0.001**	**<0.001**	**<0.001**	**0.005**	**<0.001**	**<0.001**	**<0.001**
		GmGs1	**<0.001**	**<0.001**	**<0.001**	**<0.001**	**<0.001**	**<0.001**	**<0.001**
		GmGDH1	**<0.001**	**<0.001**	**<0.001**	0.16	**0.02**	0.06	**<0.001**
		GmFd-GOGAT	**<0.001**	**<0.001**	**<0.001**	**<0.001**	**<0.001**	**<0.001**	**<0.001**
		GmNADH-GOGAT	**<0.001**	**<0.001**	**<0.001**	**0.002**	**<0.001**	0.48	**<0.001**
		GmNRT1.1	**<0.001**	**<0.001**	**<0.001**	**<0.001**	0.10	**<0.001**	0.14
Root	R1	*GmNRT2.5*	**<0.001**	**<0.001**	**<0.001**	**<0.001**	**<0.001**	**<0.001**	**<0.001**
		GmINR1	**<0.001**	**<0.001**	**<0.001**	**<0.001**	**<0.001**	**<0.001**	**<0.001**

	GmINR2	**<0.001**	**<0.001**	**<0.001**	**0.007**	**0.002**	0.10	0.31
	GmNIR	**<0.001**	**<0.001**	**<0.001**	**<0.001**	**<0.001**	**<0.001**	**<0.001**
	GmGs1	**<0.001**	**<0.001**	**<0.001**	**<0.001**	**<0.001**	**<0.001**	**<0.001**
	GmGDH1	**<0.001**	**<0.001**	**<0.001**	**<0.001**	0.96	**<0.001**	0.11
	GmFd-GOGAT	**<0.001**	**<0.001**	**<0.001**	0.18	**0.008**	**<0.001**	0.24
	GmNADH-GOGAT	**<0.001**	**<0.001**	0.61	**<0.001**	**<0.001**	**<0.001**	**<0.001**
	GmNRT1.1	**<0.001**	**<0.001**	**<0.001**	0.26	**<0.001**	0.06	0.26
	GmNRT2.5	**<0.001**	**<0.001**	**<0.001**	**<0.001**	**0.003**	**<0.001**	**<0.001**
	GmINR1	**<0.001**	**<0.001**	**<0.001**	**<0.001**	**0.03**	0.57	**0.03**
	GmINR2	**<0.001**	**<0.001**	**<0.001**	0.10	0.36	**0.004**	**<0.001**
R6	*GmNIR*	**<0.001**	**<0.001**	**<0.001**	**<0.001**	**<0.001**	**<0.001**	**<0.001**
	GmGs1	**<0.001**	**<0.001**	**<0.001**	**0.006**	**<0.001**	0.50	0.08
	GmGDH1	**<0.001**	**<0.001**	**<0.001**	**0.01**	**0.001**	0.51	0.20
	GmFd-GOGAT	**<0.001**	**<0.001**	**<0.001**	**<0.001**	**<0.001**	**<0.001**	**0.02**
	GmNADH-GOGAT	**<0.001**	**<0.001**	**0.02**	**<0.001**	**<0.001**	**<0.001**	**<0.001**

Note: Significant ($p < 0.05$) p-values are written in bold text.

Supplementary Table S5 Expression levels of nitrogen metabolism-related genes in leaves and fine roots of soybean under different treatments.

Tissue	Growth stage	Cultivar	Treatment	*GmNRT1.1*	*GmNRT2.5*	*GmINR1*	*GmINR2*	*GmNIR*	*GmGS1*	*GmGDH1*	*GmFd-GOGAT*	*GmNADH-GOGAT*
Leaf	R1	L14	LN+CC	-	0.013±0.0007f	-	0.82±0.012f	0.24±0.004f	-	0.015±0.0004f	0.11±0.005g	0.011±0.00026a
			LN+CS	-	0.020±0.0010e	-	1.27±0.017f	0.28±0.007e	-	0.018±0.0007e	0.23±0.012f	0.009±0.00057c
			LN+CR	-	0.022±0.0010de	-	1.54±0.064c	0.33±0.004d	-	0.021±0.0012cd	0.36±0.014de	0.006±0.00051ef
			MN+CC	-	0.021±0.0030e	-	1.00±0.092e	0.29±0.008e	-	0.019±0.0005e	0.24±0.023f	0.010±0.00044b
			MN+CS	-	0.025±0.0014cd	-	1.51±0.108c	0.33±0.014d	-	0.022±0.0005c	0.35±0.003e	0.006±0.00016de
			MN+CR	-	0.029±0.0015b	-	1.96±0.090b	0.39±0.006c	-	0.025±0.005b	0.45±0.020c	0.005±0.00047f
			HN+CC	-	0.026±0.0027bc	-	1.65±0.137c	0.38±0.005c	-	0.020±0.0010d	0.39±0.034d	0.007±0.00051d
			HN+CS	-	0.029±0.0022b	-	1.99±0.067b	0.47±0.010b	-	0.026±0.0005b	0.51±0.012b	0.005±0.00072f
			HN+CR	-	0.042±0.0003a	-	2.85±0.125a	0.52±0.011a	-	0.033±0.0012a	0.66±0.024a	0.004±0.00049g
		L10	LN+CC	-	0.006±0.0008f	-	0.15±0.006d	0.13±0.003g	-	0.010±0.0016e	0.08±0.005f	0.017±0.00022a
			LN+CS	-	0.010±0.0004e	-	0.22±0.007c	0.15±0.005f	-	0.014±0.0019d	0.13±0.004e	0.015±0.00069b
			LN+CR	-	0.014±0.0016d	-	0.34±0.023b	0.20±0.008de	-	0.017±0.0004bc	0.17±0.012d	0.013±0.00059c
			MN+CC	-	0.009±0.0007e	-	0.20±0.009c	0.15±0.001f	-	0.014±0.0009d	0.13±0.020e	0.015±0.00017b
			MN+CS	-	0.013±0.0026d	-	0.34±0.031b	0.19±0.009e	-	0.015±0.0011cd	0.24±0.039c	0.012±0.00047c
			MN+CR	-	0.017±0.0005c	-	0.44±0.024a	0.24±0.009c	-	0.019±0.0001b	0.29±0.004b	0.010±0.00095d
			HN+CC	-	0.015±0.0005d	-	0.34±0.033b	0.21±0.017d	-	0.014±0.0002d	0.24±0.018	0.012±0.00027c
			HN+CS	-	0.020±0.0009b	-	0.45±0.025a	0.30±0.016b	-	0.019±0.0005b	0.31±0.004b	0.010±0.00069
			HN+CR	-	0.026±0.0011a	-	0.48±0.011a	0.35±0.001a	-	0.023±0.0016a	0.38±0.005a	0.007±0.00052e
	R6	L14	LN+CC	-	0.006±0.0005f	-	0.42±0.003f	0.14±0.002e	-	0.006±0.0005g	0.08±0.003h	0.006±0.00063a
			LN+CS	-	0.008±0.0005e	-	0.65±0.021e	0.18±0.004d	-	0.011±0.0009f	0.10±0.007g	0.005±0.00039bc
			LN+CR	-	0.011±0.0005d	-	0.84±0.040d	0.22±0.007c	-	0.013±0.0004de	0.13±0.002e	0.005±0.00060de
			MN+CC	-	0.008±0.0005e	-	0.78±0.044d	0.18±0.008d	-	0.012±0.0005ef	0.11±0.005f	0.006±0.00023ab
			MN+CS	-	0.012±0.0006c	-	1.00±0.017c	0.22±0.020c	-	0.015±0.0005c	0.14±0.011d	0.004±0.00018de

			MN+CR	-	0.016±0.0009b	-	1.40±0.005b	0.30±0.025b	-	0.019±0.0005b	0.18±0.003c	0.004±0.00011e
			HN+CC	-	0.013±0.0007c	-	1.03±0.029c	0.24±0.009c	-	0.014±0.0004cd	0.15±0.005d	0.005±0.00041cd
			HN+CS	-	0.016±0.0006b	-	1.46±0.112b	0.32±0.005b	-	0.018±0.0006b	0.23±0.005b	0.003±0.00019f
			HN+CR	-	0.025±0.0005a	-	2.03±0.095a	0.39±0.013a	-	0.023±0.0020a	0.29±0.006a	0.002±0.00013g
			LN+CC	-	0.003±0.0005e	-	0.08±0.006g	0.07±0.004f	-	0.003±0.0005h	0.04±0.008f	0.012±0.00047a
			LN+CS	-	0.005±0.0007d	-	0.10±0.005fg	0.09±0.004d	-	0.005±0.0005g	0.06±0.005e	0.009±0.00082b
			LN+CR	-	0.008±0.0012c	-	0.11±0.010f	0.14±0.007c	-	0.009±0.0005e	0.09±0.005d	0.008±0.00041c
			MN+CC	-	0.005±0.0004d	-	0.10±0.002fg	0.11±0.007e	-	0.006±0.0005f	0.07±0.005e	0.007±0.00037cd
		L10	MN+CS	-	0.009±0.0005c	-	0.19±0.008d	0.14±0.004c	-	0.010±0.0008d	0.09±0.006d	0.007±0.00008de
			MN+CR	-	0.011±0.0005b	-	0.22±0.019c	0.18±0.009b	-	0.012±0.0003c	0.11±0.005c	0.006±0.00009e
			HN+CC	-	0.008±0.0004c	-	0.15±0.006e	0.14±0.003c	-	0.010±0.0005d	0.09±0.009d	0.007±0.00052de
			HN+CS	-	0.011±0.0018b	-	0.26±0.029b	0.18±0.005b	-	0.014±0.0004b	0.14±0.008b	0.005±0.00033f
			HN+CR	-	0.014±0.0006a	-	0.32±0.036a	0.21±0.005a	-	0.016±0.0003a	0.18±0.003a	0.005±0.00009f
			LN+CC	0.0022±0.000244f	0.027±0.0007h	0.0021±0.00001i	1.87±0.171f	0.08±0.013g	0.014±0.00068e	0.017±0.0005g	0.010±0.00014h	-
			LN+CS	0.0028±0.000003ef	0.061±0.0001e	0.0029±0.00007h	2.37±0.150e	0.16±0.021f	0.015±0.00018e	0.022±0.0018f	0.044±0.00268ef	-
			LN+CR	0.0051±0.000314c	0.074±0.0026d	0.0063±0.00004c	2.59±0.050e	0.48±0.001c	0.017±0.00108e	0.032±0.0008c	0.084±0.00146bc	-
			MN+CC	0.0028±0.000122ef	0.036±0.0010g	0.0035±0.00006g	2.41±0.026e	0.15±0.014f	0.014±0.00004e	0.021±0.0001f	0.027±0.00025g	-
		L14	MN+CS	0.0043±0.000187b	0.074±0.0021d	0.0046±0.00010e	2.67±0.061de	0.22±0.007e	0.022±0.00054c	0.028±0.0005d	0.058±0.00015de	-
			MN+CR	0.0065±0.000640b	0.092±0.0011b	0.0083±0.00017b	3.32±0.145c	0.74±0.011b	0.018±0.00010d	0.036±0.0016b	0.097±0.00528b	-
	R1		HN+CC	0.0034±0.000123e	0.045±0.0017f	0.0041±0.00022f	2.96±0.344d	0.21±0.001e	0.017±0.00051d	0.024±0.0007e	0.032±0.00024fg	-
			HN+CS	0.0054±0.000704c	0.081±0.0027c	0.0053±0.00010d	3.71±0.163b	0.25±0.043d	0.023±0.00050b	0.035±0.0001b	0.073±0.00127cd	-
			HN+CR	0.0080±0.000205a	0.115±0.0012a	0.0111±0.00035a	4.05±0.050a	0.83±0.009a	0.025±0.00049a	0.043±0.0003a	0.123±0.02748a	-
			LN+CC	0.0013±0.000073g	0.021±0.0012h	0.0019±0.00028g	0.33±0.047f	0.03±0.001g	0.005±0.00023g	0.012±0.0007f	0.008±0.00125i	-
			LN+CS	0.0020±0.000331ef	0.031±0.0006f	0.0023±0.00023f	0.71±0.19de	0.12±0.012e	0.006±0.00001f	0.017±0.0020e	0.020±0.00046f	-
		L10	LN+CR	0.0029±0.000201d	0.037±0.0003e	0.0047±0.00012c	0.83±0.31cde	0.20±0.009cd	0.009±0.00101e	0.022±0.0004d	0.040±0.00020d	-
Root			MN+CC	0.0019±0.000074f	0.024±0.0006g	0.0020±0.00003g	0.57±0.051ef	0.08±0.008f	0.007±0.00057f	0.014±0.0002f	0.014±0.00012h	-

		MN+CS	0.0029±0.000031d	0.041±0.0011d	0.0032±0.00002e	0.91±0.300cd	0.18±0.018d	0.008±0.00020e	0.025±0.0015c	0.029±0.00044e	-
		MN+CR	0.0044±0.000414b	0.055±0.0026b	0.0053±0.00004b	1.06±0.058c	0.25±0.033b	0.011±0.00016c	0.027±0.0020b	0.051±0.00007b	-
		HN+CC	0.0023±0.000198e	0.032±0.0001f	0.0025±0.00016f	0.98±0.063cd	0.13±0.003e	0.010±0.00080d	0.019±0.0010e	0.016±0.00100g	-
		HN+CS	0.0034±0.000356c	0.051±0.0019c	0.0042±0.00004d	1.59±0.043b	0.22±0.025c	0.015±0.00071b	0.029±0.0017b	0.042±0.00022c	-
		HN+CR	0.0058±0.000078a	0.070±0.0019a	0.0067±0.00010a	2.01±0.046a	0.31±0.015a	0.017±0.00062a	0.035±0.0008a	0.054±0.00012a	-
		LN+CC	0.0017±0.00081g	0.016±0.0021h	0.0016±0.00006f	0.80±0.030e	0.03±0.001e	0.012±0.00018g	0.0087±0.0004e	0.005±0.00106h	-
		LN+CS	0.0021±0.000145ef	0.024±0.0008g	0.0020±0.00020f	1.45±0.132d	0.04±0.005d	0.015±0.00062e	0.015±0.0013d	0.010±0.00051g	-
		LN+CR	0.0024±0.000222de	0.027±0.0013f	0.0032±0.00006de	1.73±0.198c	0.04±0.002c	0.017±0.00031d	0.022±0.0016c	0.018±0.00035ef	-
		MN+CC	0.0020±0.000094f	0.036±0.0009e	0.0021±0.00024f	1.38±0.028d	0.06±0.005d	0.013±0.00162f	0.020±0.0009c	0.015±0.00063f	-
	L14	MN+CS	0.0025±0.000093d	0.053±0.0005d	0.0028±0.00022e	1.75±0.221c	0.08±0.003c	0.017±0.0055cd	0.024±0.0015	0.020±0.00143e	-
		MN+CR	0.0029±0.000197c	0.060±0.0018c	0.0036±0.00003d	2.00±0.176c	0.12±0.005c	0.018±0.00007c	0.026±0.0020b	0.033±0.00138d	-
		HN+CC	0.0029±0.000182c	0.059±0.0025c	0.0049±0.00042c	1.94±0.110c	0.14±0.015c	0.018±0.00049c	0.023±0.0020bc	0.040±0.00093c	-
		HN+CS	0.0034±0.000319b	0.073±0.0003b	0.0056±0.00056b	2.39±0.281b	0.18±0.008b	0.023±0.00048b	0.027±0.0036b	0.051±0.00475b	-
		HN+CR	0.0042±0.000024a	0.079±0.0010a	0.0070±0.00073a	2.98±0.038a	0.27±0.017a	0.025±0.00082a	0.036±0.0032a	0.070±0.00152a	-
R6		LN+CC	0.0007±0.000178f	0.0096±0.0006d	0.0006±0.00010g	0.19±0.033f	0.01±0.003e	0.005±0.00024f	0.005±0.0004e	0.002±0.00001g	-
		LN+CS	0.0011±0.000144e	0.014±0.0004cd	0.0008±0.00019fg	0.34±0.046ef	0.01±0.001e	0.007±0.00051e	0.011±0.0005d	0.005±0.00019f	-
		LN+CR	0.0014±0.000017d	0.018±0.0034c	0.0012±0.00014ef	0.41±0.024e	0.02±0.001de	0.010±0.00022d	0.016±0.0005c	0.010±0.00382e	-
		MN+CC	0.0015±0.000022d	0.015±0.0038cd	0.0011±0.00015f	0.39±0.025e	0.02±0.001de	0.007±0.00052e	0.013±0.0003cd	0.010±0.00054e	-
	L10	MN+CS	0.0018±0.000152c	0.020±0.0016c	0.0016±0.00029e	0.83±0.113d	0.02±0.001d	0.008±0.00049e	0.016±0.0034c	0.014±0.00028d	-
		MN+CR	0.0021±0.000020b	0.030±0.0016b	0.0023±0.00037d	1.22±0.058c	0.05±0.012c	0.011±0.00095c	0.021±0.0022b	0.019±0.00189c	-
		HN+CC	0.0019±0.000068c	0.028±0.0016b	0.0029±0.00042c	1.16±0.028c	0.05±0.009c	0.011±0.00091cd	0.015±0.0019c	0.015±0.00141d	-
		HN+CS	0.0024±0.000273a	0.033±0.0051b	0.0036±0.00041b	1.58±0.016b	0.09±0.005b	0.015±0.00092b	0.018±0.0002b	0.032±0.00231b	-
		HN+CR	0.0026±0.000169a	0.052±0.0047a	0.0057±0.00022a	1.81±0.21a	0.11±0.005a	0.017±0.00032a	0.024±0.0006a	0.044±0.00201a	-

Note: Date are presented as means ± SE (n = 3). Different lowercase letters indicate significant differences among treatments within each cultivar at $p < 0.05$. Abbreviations: L10, Liaodou 10; L14, Liaodou 14; LN, low nitrogen; MN, medium nitrogen; HN, high nitrogen; CC, soybean continuous cropping soil extract; CS, soybean continuous cropping soil extract + sodium orthovanadate (Na_3VO_4); CR, soybean crop rotation soil extract. "-" indicates that data are presented in the manuscript.

Supplementary Table S6 Activities of nitrogen metabolism enzymes in leaves and fine roots of soybean under different treatments.

Tissue	Growth stage	Cultivar	Treament	NR activity	NiR activity	GS activity	GDH activity	GOGAT activity
Leaf	R1	L14	LN+CC	-	17.24±0.66e	-	0.057±0.00058a	0.32±0.012h
			LN+CS	-	19.84±2.00d	-	0.054±0.00017b	0.38±0.020g
			LN+CR	-	20.70±1.07d	-	0.053±0.00060b	0.40±0.007fg
			MN+CC	-	20.48±0.91d	-	0.051±0.00060c	0.41±0.007f
			MN+CS	-	24.19±1.12c	-	0.048±0.00099d	0.45±0.010e
			MN+CR	-	26.44±2.06bc	-	0.042±0.00111e	0.48±0.021d
			HN+CC	-	25.44±1.23bc	-	0.036±0.00080f	0.51±0.012c
			HN+CS	-	27.64±0.94ab	-	0.032±0.00115g	0.57±0.022b
			HN+CR	-	29.42±1.95a	-	0.029±0.00132h	0.61±0.017a
		L10	LN+CC	-	13.42±1.32e	-	0.061±0.00022a	0.25±0.012e
			LN+CS	-	15.99±1.88d	-	0.058±0.00063b	0.30±0.006d
			LN+CR	-	17.25±0.96d	-	0.057±0.00040bc	0.35±0.032c
			MN+CC	-	16.58±1.12d	-	0.055±0.00076c	0.36±0.024c
			MN+CS	-	17.85±1.55cd	-	0.051±0.00043d	0.43±0.003b
			MN+CR	-	20.15±1.05bc	-	0.044±0.00045e	0.45±0.025b
			HN+CC	-	18.59±1.06bcd	-	0.049±0.00050d	0.43±0.026b
			HN+CS	-	20.52±1.95b	-	0.040±0.00068f	0.51±0.011a
			HN+CR	-	23.45±0.72a	-	0.035±0.00240g	0.54±0.016a
	R6	L14	LN+CC	-	10.45±1.20f	-	0.064±0.00006a	0.21±0.012f
			LN+CS	-	11.47±0.88ef	-	0.057±0.00049b	0.25±0.034e
			LN+CR	-	13.01±0.29d	-	0.055±0.00010c	0.26±0.020e
			MN+CC	-	12.32±0.81de	-	0.053±0.00047d	0.27±0.007e
			MN+CS	-	14.48±0.75c	-	0.048±0.00150e	0.32±0.011d
			MN+CR	-	14.83±1.55bc	-	0.044±0.00061f	0.35±0.009cd
			HN+CC	-	14.72±1.97bc	-	0.042±0.00029g	0.38±0.020bc
			HN+CS	-	16.24±1.18ab	-	0.040±0.00044h	0.41±0.020ab
			HN+CR	-	17.42±1.75a	-	0.035±0.00009i	0.44±0.032a
		L10	LN+CC	-	6.66±0.52e	-	0.068±0.00089a	0.19±0.015e
			LN+CS	-	7.64±1.45de	-	0.067±0.00058a	0.20±0.010d
			LN+CR	-	8.14±1.23cd	-	0.064±0.00001b	0.22±0.020d
			MN+CC	-	8.13±1.40cd	-	0.061±0.00054c	0.23±0.027d
			MN+CS	-	8.94±0.37bc	-	0.053±0.00234d	0.28±0.007c
			MN+CR	-	9.32±0.48b	-	0.046±0.00086e	0.33±0.010b
			HN+CC	-	9.06±1.19bc	-	0.052±0.00027d	0.30±0.013c
			HN+CS	-	10.89±0.60a	-	0.043±0.00097f	0.37±0.011a
			HN+CR	-	11.91±1.34a	-	0.041±0.00057g	0.39±0.013a
Root	R1	L14	LN+CC	17.97±0.46f	10.58±0.72f	4.32±0.29f	0.031±0.00177d	-
			LN+CS	18.88±0.80def	11.95±0.19e	4.65±0.09ef	0.043±0.00169b	-

		LN+CR	19.68±1.02cde	12.33±0.24e	5.11±0.15d	0.049±0.00475a	-
		MN+CC	18.57±0.78ef	11.81±0.29e	4.72±0.14e	0.018±0.00120e	-
		MN+CS	20.28±0.18bc	13.99±0.16cd	4.91±0.19de	0.035±0.00084cd	-
		MN+CR	21.02±0.14bc	14.58±0.49bc	5.84±0.05b	0.038±0.00406c	-
		HN+CC	19.97±1.31cd	13.42±0.88d	5.19±0.13cd	0.011±0.00075f	-
		HN+CS	21.26±0.63ab	15.18±0.17ab	5.54±0.30bc	0.021±0.00446e	-
		HN+CR	22.57±0.70a	15.88±0.23a	6.78±0.24a	0.031±0.00074d	-
		LN+CC	12.11±1.06e	7.58±0.41f	3.75±0.07f	0.042±0.00132c	-
		LN+CS	16.11±1.29c	8.51±0.33e	4.21±0.12de	0.056±0.00322b	-
		LN+CR	17.98±0.60b	8.82±0.09de	4.65±0.18c	0.062±0.00200a	-
		MN+CC	13.00±0.19e	8.25±0.45ef	3.89±0.10f	0.030±0.00161e	-
	L10	MN+CS	17.06±0.29bc	9.52±0.54cd	4.49±0.25cd	0.045±0.00125c	-
		MN+CR	17.91±0.63b	10.21±0.28bc	5.10±0.12b	0.059±0.00297ab	-
		HN+CC	14.31±0.19d	9.00±0.83de	4.02±0.06ef	0.023±0.00292f	-
		HN+CS	17.48±0.49b	10.54±0.35ab	4.71±0.26c	0.035±0.00233d	-
		HN+CR	20.05±0.68a	11.13±0.42a	5.79±0.27a	0.045±0.00392c	-
		LN+CC	10.38±0.53d	7.10±0.56d	2.35±0.16e	0.020±0.00066d	-
		LN+CS	12.02±0.56c	7.09±0.57cd	2.85±0.09d	0.023±0.00078b	-
		LN+CR	14.87±0.55b	8.54±0.34bc	3.37±0.31c	0.029±0.00180a	-
		MN+CC	12.18±0.24c	8.08±0.32bcd	2.76±0.28de	0.015±0.00102ef	-
	L14	MN+CS	14.80±0.53b	8.64±0.50bc	3.57±0.15c	0.021±0.00078cd	-
		MN+CR	16.46±1.41a	9.04±0.52b	4.35±0.32ab	0.022±0.00087bc	-
		HN+CC	15.10±0.58b	8.94±0.44bc	3.73±0.32c	0.012±0.00092g	-
		HN+CS	16.77±0.50a	10.20±0.69a	4.25±0.18b	0.014±0.00006f	-
		HN+CR	17.63±0.37a	10.78±0.90a	4.71±0.10a	0.017±0.00058e	-
R6		LN+CC	6.03±0.67e	4.08±0.48e	1.46±0.10e	0.010±0.00103c	-
		LN+CS	7.22±0.58d	4.67±0.40de	2.03±0.15d	0.014±0.00060b	-
		LN+CR	8.42±0.84cd	5.22±0.19cd	2.75±0.27c	0.015±0.00055a	-
		MN+CC	7.42±1.12d	5.02±0.26d	1.94±0.18d	0.007±0.00058d	-
	L10	MN+CS	9.04±0.48bc	5.84±0.41bc	2.66±0.18c	0.009±0.00058c	-
		MN+CR	10.21±0.18ab	6.01±0.28b	3.26±0.20b	0.014±0.00054b	-
		HN+CC	8.46±0.67cd	5.91±0.34bc	2.61±0.22c	0.006±0.00053e	-
		HN+CS	9.70±0.39b	6.99±0.58a	3.22±0.20b	0.008±0.00034d	-
		HN+CR	11.21±0.42a	7.72±0.48a	3.68±0.37a	0.009±0.00074c	-

Note: Date are presented as means ± SE (n = 3). Different lowercase letters indicate significant differences among treatments within each cultivar at $p < 0.05$. Abbreviations: L10, Liaodou 10; L14, Liaodou 14; LN, low nitrogen; MN, medium nitrogen; HN, high nitrogen; CC, soybean continuous cropping soil extract; CS, soybean continuous cropping soil extract + sodium orthovanadate (Na_3VO_4); CR, soybean crop rotation soil extract. "-" indicates that data are presented in the manuscript.

Supplementary Table S7 Nitrogen content in shoots and fine roots of soybean under different treatments.

Growth stage	Cultivar	Treament	Shoot N content (2020)	Root N content (2020)	Shoot N content (2021)	Root N content (2021)
R1	L14	LN+CC	4.51±0.34i	1.44±0.09e	4.54±0.35h	1.45±0.03f
		LN+CS	7.85±0.16f	2.12±0.09	7.93±0.15e	2.04±0.11de
		LN+CR	14.00±0.44c	2.45±0.27bc	14.31±0.65b	2.46±0.32cd
		MN+CC	5.36±0.14h	1.98±0.07d	5.42±0.15g	1.97±0.17e
		MN+CS	8.80±0.23e	2.31±0.05bcd	8.82±0.17d	2.32±0.10cde
		MN+CR	15.23±0.24b	2.90±0.23a	15.56±0.63a	2.85±0.30ab
		HN+CC	6.63±0.28g	2.39±0.35bc	6.56±0.37f	2.33±0.32cde
		HN+CS	10.28±0.29d	2.54±0.19b	10.27±0.10c	2.60±0.25bc
		HN+CR	16.32±0.16a	3.21±0.12a	16.00±0.47a	3.21±0.06a
	L10	LN+CC	3.20±0.18h	0.64±0.13e	3.11±0.20e	0.62±0.17f
		LN+CS	5.63±0.08f	0.81±0.09de	5.61±0.10cd	0.92±0.21ef
		LN+CR	8.45±0.14c	1.33±0.06c	9.00±1.07c	1.31±0.16d
		MN+CC	3.50±0.01h	1.06±0.08cd	3.49±0.15e	1.04±0.10de
		MN+CS	5.54±0.18e	1.13±0.07c	6.36±0.17cd	1.14±0.11d
		MN+CR	9.45±0.08b	1.67±0.38b	9.68±2.38b	1.63±0.35bc
		HN+CC	4.61±0.17g	1.30±0.13c	4.77±0.30de	1.37±0.05cd
		HN+CS	7.48±0.14d	1.87±0.15ab	7.64±0.08bc	1.88±0.14ab
		HN+CR	11.91±0.51a	1.98±0.10a	12.04±2.03a	1.93±0.07a
R6	L14	LN+CC	3.27±0.09e	1.42±0.11f	3.22±0.16e	1.36±0.06e
		LN+CS	4.74±0.51d	1.66±0.05ef	4.63±0.53d	1.59±0.08d
		LN+CR	5.30±0.24c	2.05±0.14cd	5.32±0.26c	2.06±0.12cd
		MN+CC	4.62±0.24d	1.93±0.34de	4.81±0.32d	1.95±0.37d
		MN+CS	5.23±0.15c	2.30±0.08bc	5.40±0.29c	2.28±0.08bc
		MN+CR	6.51±0.20b	2.52±0.14ab	6.44±0.23b	2.50±0.08ab
		HN+CC	5.43±0.14c	2.17±0.11cd	5.46±0.10c	2.19±0.12cd
		HN+CS	6.91±0.07b	2.60±0.23ab	6.74±0.06b	2.68±0.18a
		HN+CR	7.53±0.08a	2.76±0.14a	7.56±0.03a	2.76±0.14a
	L10	LN+CC	2.51±0.17e	0.47±0.17e	2.38±0.28e	0.50±0.16d
		LN+CS	3.30±0.08d	0.73±0.14de	3.30±0.13d	0.74±0.14c
		LN+CR	3.64±0.34d	1.14±0.10c	3.34±0.32d	1.14±0.16b
		MN+CC	3.34±0.20d	0.67±0.23de	3.38±0.17d	0.71±0.24cd
		MN+CS	4.30±0.01c	1.17±0.14c	4.33±0.01c	1.25±0.16b
		MN+CR	5.36±0.08b	1.46±0.06b	5.41±0.15b	1.49±0.11a
		HN+CC	4.33±0.14c	0.93±0.15cd	4.36±0.11c	0.90±0.22bc
		HN+CS	5.68±0.34b	1.51±0.05ab	5.65±0.42b	1.52±0.09a
		HN+CR	6.55±0.17a	1.74±0.10a	6.53±0.22a	1.74±0.15a
R8	L14	LN+CC	17.74±1.16h	0.89±0.18g	18.00±4.15g	0.89±0.19g
		LN+CS	21.49±1.03g	1.24±0.11f	20.41±2.59fg	1.21±0.10f

	LN+CR	25.08±0.64f	1.52±0.17e	25.50±0.85f	1.48±0.20e
	MN+CC	22.84±1.84fg	1.33±0.14ef	23.27±3.83f	1.33±0.20ef
	MN+CS	28.89±1.94e	1.99±0.13d	31.83±2.39e	1.97±0.11d
	MN+CR	36.66±0.72d	2.13±0.12cd	36.38±2.33d	2.14±0.16cd
	HN+CC	44.07±1.70c	2.27±0.16bc	45.06±2.16c	2.28±0.15bc
	HN+CS	49.97±2.54b	2.48±0.14b	49.18±3.22b	2.47±0.11b
	HN+CR	56.76±1.01a	2.79±0.06a	54.93±2.06a	2.79±0.03a
	LN+CC	14.07±1.59f	0.34±0.06g	14.87±1.42f	0.31±0.05f
	LN+CS	16.84±1.32ef	0.59±0.12f	16.62±1.83f	0.55±0.12f
	LN+CR	18.31±2.16e	0.95±0.19e	18.90±4.81ef	0.94±0.20e
	MN+CC	20.87±1.42e	1.10±0.15e	22.26±2.31e	1.11±0.17e
L10	MN+CS	25.86±1.85d	1.65±0.04d	25.61±1.06d	1.64±0.07d
	MN+CR	27.13±1.05cd	1.85±0.06cd	26.17±2.71d	1.84±0.07cd
	HN+CC	30.46±1.89c	2.03±0.14bc	30.67±0.82c	2.01±0.16bc
	HN+CS	40.71±3.64b	2.16±0.21ab	40.69±1.75b	2.16±0.26ab
	HN+CR	47.87±3.60a	2.34±0.14a	49.04±2.21a	2.35±0.12a

Note: Date are presented as means ± SE (n = 3). Different lowercase letters indicate significant differences among treatments within each cultivar at $p < 0.05$. Abbreviations: L10, Liaodou 10; L14, Liaodou 14; LN, low nitrogen; MN, medium nitrogen; HN, high nitrogen; CC, soybean continuous cropping soil extract; CS, soybean continuous cropping soil extract + sodium orthovanadate (Na_3VO_4); CR, soybean crop rotation soil extract.

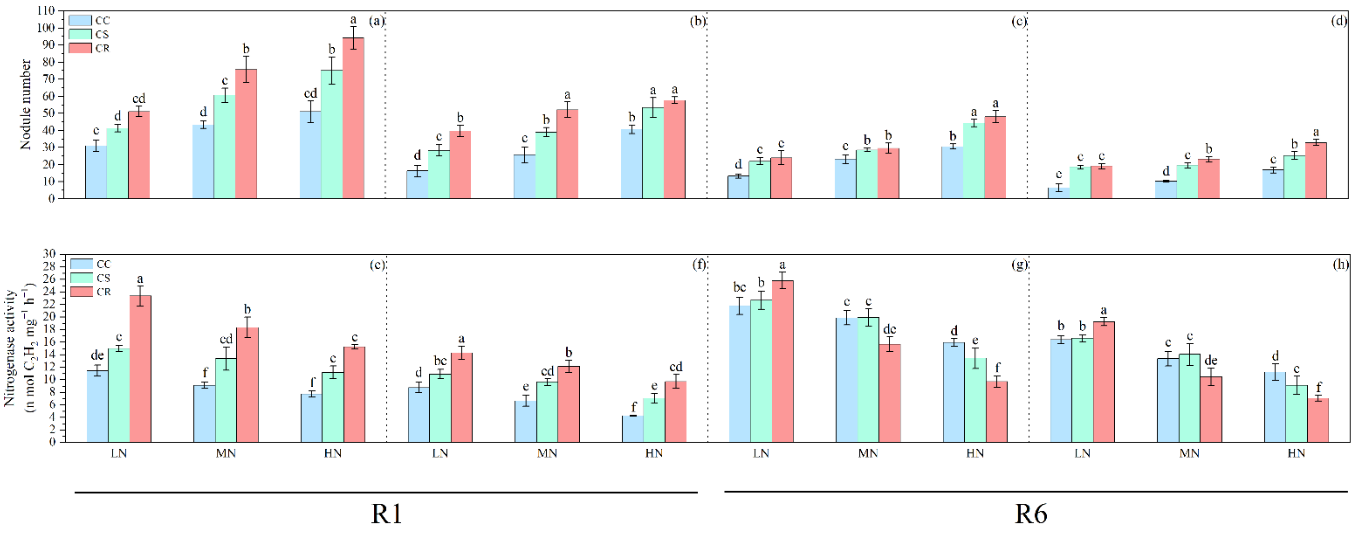

Supplementary Figure S1. Effects of nitrogen levels on nodule number and nitrogenase activity of soybean cultivars with different continuous cropping tolerance in 2021. Different lowercase letters above bars indicate significant differences among treatments within each cultivar at $p < 0.05$. Abbreviations: R1, initial flowering growth stage; R6, full seed growth stage; LN, low nitrogen; MN, medium nitrogen; HN, high nitrogen; CC, soybean continuous cropping soil extract; CS, soybean continuous crop-ping soil extract + sodium orthovanadate (Na_3VO_4); CR, soybean crop rotation soil extract. Panels a, b, e, and f represent the R1 growth stage; panels c, d, g, and h represent the R6 growth stage. Panels a, c, e, and g represent Liaodou 14 (L14); panels b, d, f, and h represent Liaodou 10 (L10).

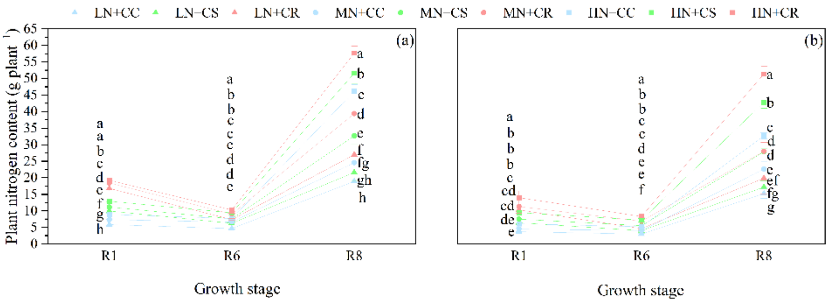

Supplementary Figure S2. Dynamic changes in total nitrogen accumulation at different growth stages of soybean cultivars differing in continuous cropping tolerance under varying nitrogen levels in 2021. Different lowercase letters in the figure indicate significant differences among treatments at the same growth stage at $p < 0.05$. Abbreviations: R1, initial flowering growth stage; R6, full seed growth stage; R8, full maturity growth stage; LN, low nitrogen; MN, medium nitrogen; HN, high nitrogen; CC, soybean continuous cropping soil extract; CS, soybean continuous cropping soil extract + sodium orthovanadate (Na_3VO_4); CR, soybean crop rotation soil extract. Panel a represents Liaodou 14 (L14); panel b represents Liaodou 10 (L10).

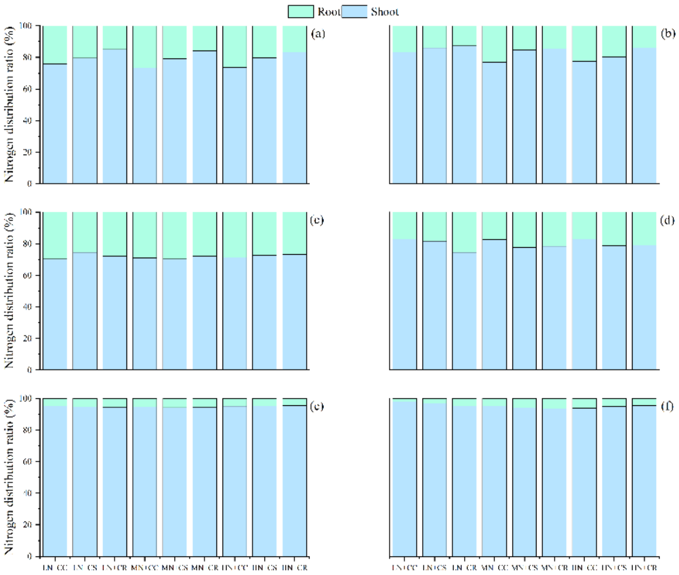

Supplementary Figure S3. Nitrogen distribution ratio between shoots and roots of soybean cultivars differing in continuous cropping tolerance under varying nitrogen levels in 2021. Abbreviations: LN, low nitrogen; MN, medium nitrogen; HN, high nitrogen; CC, soybean continuous cropping soil extract; CS, soybean continuous cropping soil extract + sodium orthovanadate (Na_3VO_4); CR, soybean crop rotation soil extract. Panels a and b represent the initial flowering growth stage (R1); panels c and d represent the full seed growth stage (R6); panels e and f represent the full maturity growth stage (R8). Panels a, c, and e represent Liaodou 14 (L14); panels b, d, and f represent Liaodou 10 (L10).
